# Redox-Active Quinazolinone Thioamide Ag(I) Complexes with Potent Antibacterial Activity: Mechanistic Insights and Hydrogel-Enhanced Efficacy

**DOI:** 10.3390/molecules30204071

**Published:** 2025-10-13

**Authors:** Eleni Ioanna Tzaferi, Despoina Varna, Igor V. Esarev, Konstantina Kavaratzi, Antonios G. Hatzidimitriou, Rigini Papi, Ingo Ott, Panagiotis A. Angaridis

**Affiliations:** 1 Laboratory of Inorganic Chemistry, Department of Chemistry, Aristotle University of Thessaloniki, 54124 Thessaloniki, Greece; eleantzaf@gmail.com (E.I.T.); varndesp@chem.auth.gr (D.V.); kkavaratzi@chem.auth.gr (K.K.); hatzidim@chem.auth.gr (A.G.H.); 2 Institute of Medicinal and Pharmaceutical Chemistry, Technische Universität Braunschweig, 38106 Braunschweig, Germany; i.esarev@tu-bs.de; 3 Laboratory of Biochemistry, Department of Chemistry, Aristotle University of Thessaloniki, 54124 Thessaloniki, Greece; rigini@chem.auth.gr

**Keywords:** silver(I) complexes, phosphines, heterocyclic thioamides, alginate-based hydrogels, antibacterial activity, TrxR inhibition, GR inhibition, redox activity

## Abstract

The antibacterial properties of Ag(I) coordination compounds are well documented; however, their effectiveness is highly dependent on the choice of appropriate ligands, and it is frequently hindered by their low water solubility and limited bioavailability. Herein, six new Ag(I) complexes incorporating the quinazolinone thioamide mqztH (=2-mercapto-4(3H)-quinazolinone) and phosphine co-ligands were synthesized and investigated for their antibacterial activity. In vitro activity assays against *Escherichia coli* (*E. coli*) and *Staphylococcus aureus* (*S. aureus)* bacterial strains revealed that all complexes selectively inhibited *S. aureus* bacterial growth. Structure–activity relationship analysis showed that monodentate PPh_3_ co-ligands play a key role in enhancing the antibacterial efficacy of their complexes. Notably, complex [AgCl(mqztH)(PPh_3_)_2_] (**1**) exhibited broad-spectrum activity, with IC_50_ values of 4.2 ± 1.4 μg mL^−1^ (4.9 μΜ) for *S. aureus* and 63 ± 1.9 μg mL^−1^ (75 μΜ) for *E. coli* bacteria. To improve solubility and antibacterial activity, complex **1** was encapsulated in barium alginate (**BaAlg**) matrices to form hydrogel-based drug delivery formulations **[1]@BaAlg**. The synthesized formulations retained the bactericidal effect of the complex, achieving comparable activity at concentrations lower by an order of magnitude compared to complex **1** in free form. Combined with the demonstrated high biocompatibility of complex **1** toward L929 normal eukaryotic cells, as well as the biocompatible nature of the alginate matrix, these findings underscore the strong potential of the complex **1**-loaded hydrogel formulations for further investigation and development as effective antibacterial drug platforms. Mechanistic studies confirmed the redox-active nature of complex **1** and its potential to inhibit the function of glutathione reductase (GR) and thioredoxin reductase (TrxR) at low concentrations, suggesting the interference with bacterial redox homeostasis as a relevant mechanism of bioactivity.

## 1. Introduction

Antibiotic-resistant bacteria are gradually recognized as a major health issue globally. According to recent literature reports, antimicrobial resistance was responsible for almost 5 million deaths worldwide in 2019 [1]. Moreover, if no effective measures are taken, the number of deaths is expected to dramatically increase in coming decades, with severe consequences on human health and world economy [2]. This risk mainly arises from the excessive overapplication and misuse of antibiotics, which have resulted in the development of resistant bacterial strains [3,4]. As an increasing number of currently available therapies lose their effectiveness, there is a need for the development of new antimicrobial agents that can bypass resistance mechanisms and target drug-resistant microbes effectively [5].

Scientists are currently exploring several innovative biotechnology approaches to combat antimicrobial resistance [6,7,8]; however, these often face challenges related to molecular stability, mechanisms for delivery, and approval procedures [9,10]. Interestingly, metal-based drugs and, specifically, Ag(I) compounds seem particularly promising, as they combine antimicrobial activity properties with relatively simple drug development process, owing to their long history of use as antimicrobial agents [11]. This makes them an attractive connection between new therapeutic agents and fast practical application in the real world. In fact, some Ag(I) compounds are already used as active components in pharmaceutical products [12]. These include: AgNO_3_ solutions for the treatment of eye infection [13], Ag(I) sulfadiazine for the prevention and treatment of wound infections [14], and Ag(I)-impregnated contact lens cases to prevent bacterial colonization [15]. In addition, recent literature reports have documented the potential of Ag(I) coordination compounds bearing certain ligands as antimicrobial agents, with some of them found to exhibit broad-spectrum efficacy against Gram (+) and Gram (−) bacteria [16], low cytotoxicity against human cells [17], and reduced potential for inducing resistance [18,19,20,21,22]. These reports have prompted further research efforts in the field, aiming at the development of new Ag(I) complexes with enhanced antibacterial activity, as alternatives to conventional antibacterial agents [23,24,25,26,27].

Antibacterial activity of Ag(I) complexes stems from their multi-modal mechanism of action involving: (a) bacterial membrane interaction (particularly the periplasm), (b) DNA binding, and (c) inhibition of enzymes that contain thiol groups and are responsible for cellular redox homeostasis [28]. Specifically, acting as enzyme inhibitors, e.g., of glutathione reductase (GR) and thioredoxin reductase (TrxR), Ag(I) complexes can perturb redox equilibria within cells, ultimately influencing cell viability [29]. These multi-faceted interactions impacting several key biomolecular pathways result in enhanced therapeutic effects for Ag(I) complexes, rendering them strong candidates to address antimicrobial resistance.

Ag(I) complexes with heterocyclic thioamide ligands, either in their neutral form (L^NH^S) or their anionic form (L^N^S^−^), have shown strong potential as inhibitors of bacterial growth, although their precise bioactivity mechanisms remain unclear and requires further investigation. Their effectiveness is greatly affected by their individual structural characteristics, such as total charge, number of Ag(I) ions, saturation degree of Ag(I) coordination sphere, as well as nature and type of donor atoms, coordination properties and functional groups of their ligands. We recently reported that cationic, coordinatively unsaturated, mononuclear complexes of the general types [Ag(L^NH^S)(PPh_3_)_2_]^+^ and [Ag(L^NH^S)(P^^^P)]^+^, where P^^^P = diphosphine, demonstrate exceptional activity, particularly against *S. aureus* bacterial strain, likely due to the high affinity of Ag(I) ions for S-containing bacterial proteins [30].

Despite their encouraging efficacy, most of the bioactive Ag(I) complexes suffer from low water solubility, which leads to poor bioavailability that hinders their clinical application [31]. To overcome these shortcomings and improve pharmacokinetics, new strategies for the efficient delivery of the complexes have emerged. These involve their encapsulation in polymeric nanoplatforms, films, as well as hydrogels [32,33]. Hydrogels are composed of crosslinked three-dimensional polymer chain networks which are hydrophilic in nature, and they can absorb large amounts of water without losing their structural integrity. Due to their high water content, permeability, and elastic and viscous behavior, they closely resemble living tissue and, therefore, they are extensively used in biomedicine, e.g., drug release under controlled conditions [34]. Among different hydrogel-forming materials, naturally occurring polysaccharides have attracted much attention over the last few years, owing to their excellent mechanical behavior, biocompatibility, and biodegradability [35,36]. An example is sodium alginate (**Alg**) which is composed of β-D-mannuronic acid and α-L-guluronic acid units and is able to form highly stable hydrogels via ionic crosslinking with bivalent cations in aqueous solutions [37].

As a continuation of our recent work on Ag(I) complexes with various thioamide ligands [24,28,30,33], herein we present a series of Ag(I) complexes with 2-mercapto-4(3H)-quinazolinone (mqztH), in its neutral or deprotonated form, and phosphine co-ligands, and study their in vitro activity against the *E. coli* and *S. aureus* bacterial strains. MqztH as ligand appears to be a very attractive one, as it embeds the coordinating thioamide moiety into the quinazolinone scaffold, a pharmacologically relevant motif that is known for antimicrobial, anticancer, and anti-inflammatory properties [38,39,40,41]. Aiming to enhance biocompatibility and pharmacokinetics of the Ag(I) complexes, **Alg**-based hydrogel formulations were prepared and studied as drug-delivery systems against the *S. aureus* bacterial strain. Finally, in vitro bioactivity assays against the bacterial redox active enzymes GR and TrxR, as well as electrochemical studies, were conducted to gain an insight into possible bioactivity mechanisms of this group of complexes. The results presented herein contribute to the growing field of Ag(I) complexes with antibacterial properties and offer an advance in developing efficient and biocompatible antibacterial agents and formulations.

## 2. Results and Discussion

### 2.1. Synthesis, Crystal Structures and Characterization of Complexes **1**–**6**

**Scheme 1:**
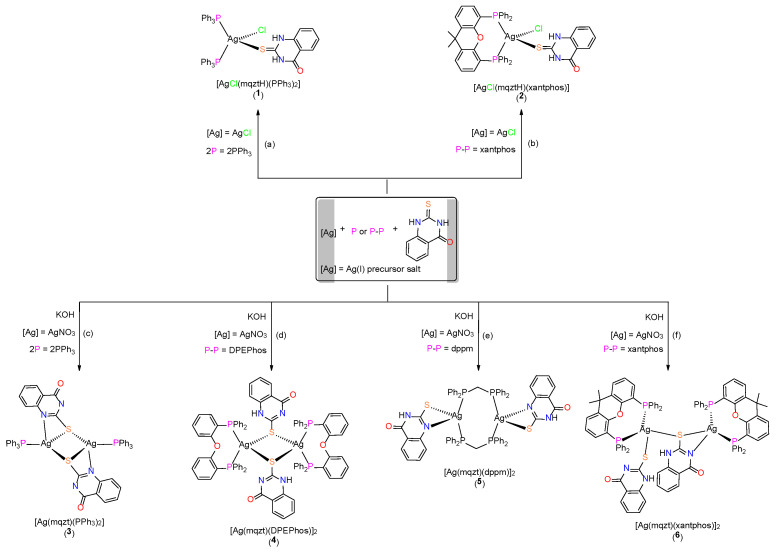
Syntheses of complexes **1**–**6**. Experimental conditions: (**a**,**b**) CH_3_CN, 50 °C, 2 h; (**c**–**f**) CH_2_Cl_2_, r.t., 2 h.

A group of heteroleptic Ag(I) complexes bearing the heterocyclic thioamide mqztH, in its neutral or deprotonated form, and phosphine co-ligands was synthesized. Structural diagrams of the complexes (**1**–**6**) as well as brief descriptions of their syntheses are provided in Scheme 1 (detailed experimental procedures are described in S1.2 in the Supplementary Materials).

All complexes were synthesized by following a standard one-pot/two-step synthetic procedure. Mononuclear complex [AgCl(mqztH)(PPh_3_)_2_] (**1**) was obtained by treatment of AgCl with PPh_3_ in 1:2 mole ratio followed by addition of 1 equiv of mqztH in CH_3_CN/MeOH at 50 °C for 2 h. The synthesis of the structurally analogous [AgCl(mqztH)(xantphos)] (**2**) was achieved in a similar way, using 1 equiv of xantphos instead of 2 equiv of PPh_3_. Addition of the thioamide ligand in its deprotonated form, K^+^(mqzt)^−^, to a mixture of AgNO_3_ and PPh_3_ in CH_2_Cl_2_ at room temperature resulted in the synthesis of the binuclear complex [Ag(mqzt)(PPh_3_)]_2_ (**3**). The analogous reactions using equimolar amounts of the diphosphines DPEPhos, dppm and xantphos instead of PPh_3_, and under the same experimental conditions, afforded the binuclear complexes [Ag(mqzt)(DPEPhos)]_2_ (**4**), [Ag(mqzt)(dppm)]_2_ (**5**) and [Ag(mqzt)(xantphos)]_2_ (**6**), respectively. All complexes were isolated in the form of colourless microcrystalline solids after filtration of their corresponding reaction mixtures and subsequent crystallization of the filtrates. They were found to be stable in air, either in the solid state or solutions in organic solvent for long periods of time.

**Figure 1:**
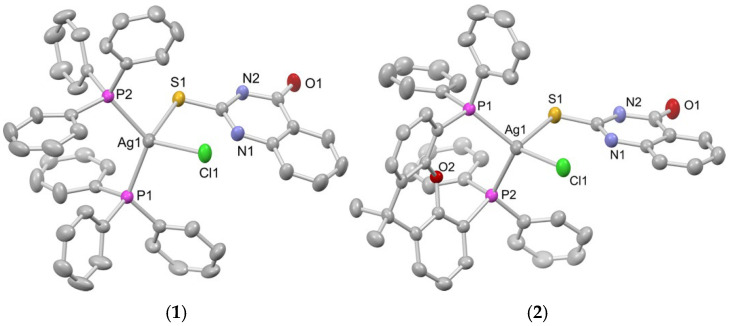
Views of the molecular structures of complexes **1** and **2** with atoms drawn as thermal ellipsoids at the 35% probability level. Hydrogen atoms are omitted for clarity.

**Figure 2:**
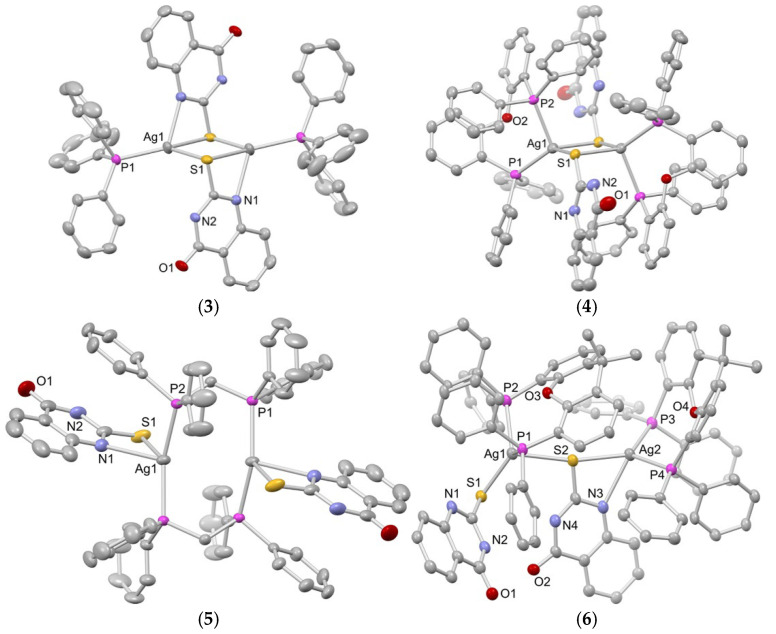
Views of the molecular structures of complexes **3**–**6** with atoms drawn as thermal ellipsoids at the 35% probability level. Hydrogen atoms are drawn as spheres of arbitrary radii.

The molecular structures of complexes **1**–**6** were determined in the solid state using single-crystal X-ray diffraction analysis. Key crystallographic data, along with details of data collection and structure refinement, are presented in Table S1. Views of the crystal structures of the complexes are illustrated in Figure 1 and Figure 2 as well as Figures S1–S6, while selected bond lengths and angles are provided in Tables S2–S7.

Mononuclear complexes **1** and **2**, [AgCl(mqztH)(PP)], with PP = 2PPh_3_ for complex **1** and xantphos for complex **2**, respectively (crystallized as **1**∙CH_3_CN and **2** CH_3_CN), exhibit comparable molecular structures, each featuring a central Ag(I) ion in a tetrahedral coordination environment. This is defined by two P atoms – originating either from two monodentate PPh_3_ ligands or a chelating bidentate xantphos ligand—the exocyclic S atom of a neutral heterocyclic thioamide ligand mqztH, and a Cl^−^ ion. Bond angles around the metal centers deviate from ideal tetrahedral values, with notable examples including P2–Ag–S angles of 97.84(5)° in complex **1** and 99.55(4)° in complex **2**, and S–Ag–Cl angles of 98.63(4)° and 101.38(3)°, respectively. The Ag–P bond lengths are comparable between the two complexes, with 2.484(2) and 2.466(1) Å for complex **1**, and 2.469(1) and 2.505(1) Å for complex **2**. Similarly, their Ag–S bond lengths are measured at 2.764(2) and 2.664(1) Å, respectively. These structural characteristics align well with those reported for structurally related Ag(I) complexes in the literature [42,43]. In both crystal structures, intramolecular hydrogen bonding interactions are observed between the NH group of their mqztH moieties and the Cl^−^ ligands. These interactions are evidenced by the tilted orientation of the thioamide ring toward the Cl^–^ ion and by the short N⋯Cl interatomic distances of 3.079 and 3.107 Å, respectively (Figures S1 and S2 and Tables S2 and S3).

Complex [Ag(mqzt)(PPh_3_)]_2_ (**3**) (crystallized as **3**∙2CH_2_Cl_2_) is a symmetrical binuclear compound in which two Ag(I) ions are held in proximity by two heterocyclic thioamidate mqzt^−^ ligands. Each thioamidate ligand exhibits simultaneously multiple coordination modes: (a) serving as a bridging ligand through its S atom between the two metal centers and forming a central Ag_2_(μ-S)_2_ core; (b) coordinating via its S atom to a single Ag(I) ion; and (c) acting as an S,N-chelating ligand to the other Ag(I) ion. Additionally, each Ag(I) ion is coordinated by a PPh_3_ ligand, overall resulting in a PS_2_N coordination environment around each metal center. The multiple coordination modes of the thioamidato ligands lead to significant distortions from the ideal tetrahedral geometry of the two metal centers, which is evidenced by the pertinent bond angles S–Ag–N (=60.50(9)° and 100.71(9)°), P–Ag–N (=116.21(9)°), P–Ag–S (=131.09(4) and 133.03(4)°) and S–Ag–S (=92.23(4)°). The Ag–N and Ag–P bond lengths are 2.430(4) and 2.389(1) Å, respectively, and they are unexceptional [24]. The Ag–S bond lengths of the central Ag_2_(μ-S)_2_ core are at 2.557(1) Å and 2.776(1) Å, resulting in a rhombic-like geometry. The Ag⋯Ag distance within the Ag_2_(μ-S)_2_ core is 3.701 Å (Table S4), which is more than the sum of the van der Waals radii of two neighbouring Ag atoms (~1.7 Å), indicating the absence of any intermetallic bonding interactions.

Complex [Ag(mqzt)(DPEPhos)]_2_ (**4**) (crystallized as **4**∙C_6_H_14_) also exhibits a symmetrical binuclear structure resembling that of complex **3**, but with notable differences in coordination behavior and arrangement of ligands. In this case, each mqzt^−^ ligand functions solely in a bridging mode through its S atom, connecting the two Ag(I) ions and forming a central Ag_2_(μ-S)_2_ core. The coordination sphere of each Ag(I) ion is completed by the two P atoms of a bidentate DPEphos ligand, overall resulting in a P_2_S_2_ coordination environment around each metal center. Structurally, the two mqzt^−^ ligands are located on opposite sides of the central Ag_2_(μ-S)_2_ core, with their fused ring planes oriented nearly perpendicular to the core itself. Bond angles around Ag(I) ions vary from 90.10(4)° (for P2–Ag1–S1) to 132.06(5)° (for P1–Ag1–S1), suggesting significant deviations from the ideal tetrahedral coordination geometry. The P–Ag–P bond angles are close to the natural bite angle of DPEphos (~104°), while the Ag–P bond lengths are unexceptional. The Ag–S bond lengths of the central Ag_2_(μ-S)_2_ core are not equal (2.620(2) Å and 2.638(2) Å), resulting in a distorted rhombic geometry. Noticeably, the interatomic Ag⋯Ag distance is shorter than that in **3** at 3.343(1) Å (Table S5), and it indicates the presence of weak intermetallic bonding interactions.

Complex [Ag(mqzt)(dppm)]_2_ (**5**), although also being a symmetrical binuclear compound, adopts a fundamentally different structural framework compared to complexes **3** and **4**. In this complex, the two Ag(I) ions are bridged by two diphosphine (dppm) ligands, which span opposite coordination sites on each Ag center. This arrangement results in the formation of a nearly planar Ag_2_P_4_C_2_ six-membered ring that defines the core of the structure. Additionally, each Ag(I) ion is coordinated by an mqzt^−^ ligand, which binds in a bidentate chelating fashion via its S and N atoms. These ligands occupy the external coordination sites of the Ag_2_P_4_C_2_ ring, with their fused ring systems oriented nearly perpendicular to the P–Ag–P axes. This ligand arrangement introduces geometric structural rigidity, while contributing to a unique spatial organization that sets complex **5** apart from the abovementioned binuclear complexes **3** and **4**. Bond angles around the metal centers show deviations from those of the ideal tetrahedral coordination, due to the restrictions imposed by the geometrical characteristics of the donor atoms of the ligands. For example, the P–Ag–P and N–Ag–S bond angles are at 149.29(3)° and 60.97(8)°, respectively. Interestingly, the P–Ag–P angles are found to be slightly larger than that those observed in complexes with similar structures, such as [Ag_2_(dppm)_2_(NO_2_)_2_] showing P–Ag–P angles of 148.6(1)° [44]. The Ag–P, Ag–N and Ag–S bond distances are found at 2.410(1)/2.452(1), 2.520(3) and 2.665(1) Å, respectively. Finally, the complex displays an interatomic Ag⋯Ag distance of 3.557(1) Å (Table S6), which is longer than the sum of van der Waals radii of two Ag(I) (3.44 Å), indicating the absence of intermetallic bonding interactions.

Finally, complex [Ag(mqzt)(xantphos)]_2_ (**6**) (crystallized as **6**∙CH_2_Cl_2_) is a binuclear compound adopting an unsymmetrical structural framework. The two Ag(I) centers are bridged by the exocyclic S atom of a thioamide ion, which also coordinates to one of the two Ag(I) ions via its N atom, forming an S,N-chelating coordination mode. In addition, an S-bound thioamidato ligand is coordinated to the second Ag(I) center. Each Ag(I) ion completes its coordination spheres by the P atoms of a chelating xantphos ligand. As a result, the two metal centers exhibit distinct coordination environments, namely P_2_SN and P_2_S_2_. This unsymmetrical coordination pattern and unique orientation of the ligands in complex **6** are likely the result of the steric and conformational rigidity of the xantphos ligand. In contrast, the homologous complex **3**, featuring the more flexible and less sterically demanding diphosphine DPEPhos ligand, adopts a symmetrical molecular architecture. Significant deviations from the ideal tetrahedral coordination geometry are observed at both metal centers, as indicated by the P2–Ag1–S2 and P3–Ag2–S2 bond angles of 93.48(8)^o^ and 129.66(12)^o^, respectively. The Ag–P and Ag–S bond lengths, averaging at 2.476 and 2.592 Å, respectively, are consistent with those reported for Ag(I) complexes containing analogous thioamidato diphosphine donors [30]. Finally, the structure of complex **6** was found to be stabilized by intramolecular hydrogen-bonding interactions developed between the NH and C=O groups of the bridging thiomidate ligand with the S atom and the NH group, respectively, of the terminally coordinated thioamidate ligand, with the pertinent N(H)⋯S and N(H)⋯O distances found at 3.349 and 3.039 Å, respectively (Table S7).

**Figure 3:**
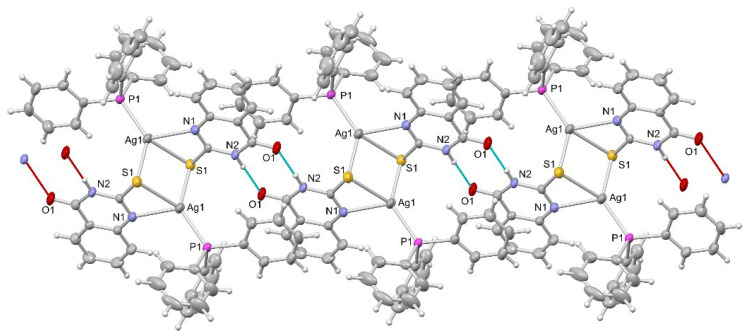
View of the intermolecular N–H⋯O hydrogen-bonding interactions (blue-color dotted lines) developed among neighboring molecules of complex **3** in the solid state, giving rise to an infinite chain (red-color dotted lines at the edges denote interactions with neighboring molecules not shown in the figure). Atoms are presented as thermal ellipsoids at the 35% probability level. Hydrogen atoms are shown as spheres of arbitrary radius.

A detailed analysis of the packing of the complexes in the solid-state reveals the formation of extended architectures in the cases of complexes **1**–**3** and **5**, as illustrated in Figure 3 and Figures S7–S10. These are formed mainly through intermolecular hydrogen bonding interactions involving the exocyclic S atoms and either the NH or the C=O group of their thioamide or thioamidate ligands. As shown in the views of the crystal lattice of complex **3** in Figure 3 and Figures S9, hydrogen bonding interactions between the NH group of the mqzt^−^ ligand in one molecule and the O atom of the C=O group of the mqzt^–^ ligand in an adjacent molecule (in combination with weak π-π interactions) result in the formation of infinite molecular chains. The pertinent N–H⋯O distances are at 2.897 Å, consistent with similar values reported for related Ag(I) thioamidate complexes [28]. Analogous infinite molecular chains are formed in the crystal structure of complex **5**, stabilized through N(H)⋯S (=3.260 Å) intermolecular interactions (Figure S10). In contrast, NH⋯O intermolecular interactions in complexes **1** and **2**, with interatomic distances of 2.825 and 2.857 Å (Figures S7 and S8), give rise to oligomeric molecular architectures (molecular pairs). These findings collectively highlight the ability of complexes to engage in hydrogen-bonding interactions via the NH or C=O groups of their thioamide or thioamidate ligands, suggesting potential interactions with biological substrates or targets that may influence their bioactivity.

Fourier-transform infrared (FTIR) spectra of complexes **1**–**6** confirmed their identity as microcrystalline solids isolated from their respective reaction mixtures. Their FTIR spectra in the 4000–400 cm^−1^ range show absorption bands attributed to vibrations of the heterocyclic thioamide and the respective phosphine ligands (Table S9). Characteristic absorption bands associated with vibrations of the thioamide group of the heterocyclic thioamide are of high diagnostic value, as they undergo clear shifts, either to lower or higher frequencies with respect to those of the ligand in its free form. For example, the FTIR spectrum of free mqztH exhibits an absorption band at 1567 cm^−1^, attributed to the C=N bond vibration. In the FTIR spectrum of complex **1**, this band is present but shifted to 1552 cm^−1^, because of the thioamide coordination to the metal center. In addition, the absorption band of free mqztH at 1269 cm^−1^, attributed to the thioamide group vibration, slightly shifts to a lower frequency at 1268 cm^−1^ in the spectrum of complex **1**. This shift aligns with the electron transfer $H\vec{\ddot{N}\text{-}}C\vec{=S}:\to \mathrm{Ag}$ that occurs when the thioketo form of the thioamide coordinates to the metal center through its exocyclic S atom. Similarly, the absorption bands at 1169 and 762 cm^−1^ in the spectrum of free mqztH, which are linked to the C=S bond vibration, are shifted to 1095 and 692 cm^−1^, respectively, in the spectrum of complex **1**. Analogous absorption band shifts associated with the thioamide or thioamidate group (Table S9) can be recognized in the FTIR spectra of complexes **2**–**6**, which corroborate their identity and are consistent with the molecular structures of the complexes determined by X-ray diffraction.

The ^1^H NMR spectra of complexes **1**–**6** were recorded to assess their structural integrity in solution, specifically in DMSO-d_6_, a solvent commonly used in biological and pharmaceutical studies for compounds with poor water solubility. All complexes exhibited H signals that correspond to their respective ligands (Figures S11–S16), which were shifted relative to those of the free ligands, reflecting coordination to the metal center. For example, in the ^1^H-NMR spectrum of complex **1**, distinct singlet and doublet signals are observed in the downfield region at 8.99 ppm and 8.06–8.05 ppm, respectively. These signals are attributed to the H’s of the two NH groups of the mqztH ligand, indicating coordination of the thioamide to the metal center through its exocyclic S atom. The observed chemical shifts are likely due to deshielding effects caused by intramolecular hydrogen bonding between the NH groups with the Cl^−^ ligand and the CO group of the mqztH ligand. Additionally, in the region of 7.75–7.74 ppm and 7.67–7.65 ppm, the aromatic H’s of the thioamide ligand appear as doublets or multiplets. The signals of the H’s of the two PPh_3_ ligands appear as partially resolved multiplets in the region of 7.44–7.41, 7.32–7.31 and 7.26–7.23 ppm, corresponding to the m-H, p-H and o-H of their phenyl groups. Complex **2**, analogous to complex **1**, exhibits two singlets at 12.75 and 12.47 ppm, attributed to the NH and N^2^H protons of mqztH. The aromatic protons of the thioamide moiety appear as doublets or multiplets in the range of 7.92–7.70 ppm. In a slightly higher field, between 7.40–7.29 ppm, multiplet signals corresponding to the phenyl protons of the xantphos ligand are observed. The protons on the diphosphine backbone give rise to peaks at 6.92–6.90 ppm (triplet), 6.78–6.79 ppm (doublet), and 6.58–6.56 ppm (multiplet). Additionally, a singlet at 1.56 ppm, corresponding to the six methyl protons of the xantphos ligand, suggests dynamic behavior of complex **2** in solution. The analogous to **1**, complex **2**, displays two singlet peaks at 12.75 and 12.47 ppm corresponding to the H’s of N^1^H and N^2^H of the mqztH. Signals of the aromatic H’s of the thioamide are observed as doublets or multiplets in the region 7.92–7.70 ppm whereas, in higher field regions of 7.40–7.29 ppm, the signals of H’s of the phenyl groups of xantphos appear as multiplets. H’s of diphosphine’s backbone give signals at 6.92–6.90, 6.78–6.79 and 6.58–6.56 ppm as triplet, doublet and multiplet peaks. A singlet at 1.56 ppm corresponds to the 6H’s of the CH_3_ froups of xantphos, indicating a dynamic behavior of **2** in solution. Binuclear complex **3** bears the thioamide in its protonated form, thus only one singlet peak at 12.05 ppm corresponding to the H, N^2^H is observed. Signals of the aromatic H’s of mqzt^-^ appear as multiplets at 7.59–741 ppm whereas o-H, p-H and m-H of PPh_3_ show multiple signals in the region 7.40–7.28 ppm. The spectrum of **4** displays similar signals to **3**, with a broad singlet at 12.09 ppm corresponding to the NH groups of the thioamide. The other four H’s of mqztH display multiple signals at 8.17–8.08 ppm whereas in 7.85–7.00 signals of the dppm are also observed as multiplets. An additional singlet corresponding to the H of dppm’s backbone appeared at 3.61 ppm. The binuclear complex **5** exhibits a singlet at 11.74 which is ascribed to the NH of mqzt and multiple signals at the aromatic region corresponding to the H’s of DPEphos. Likewise, complex **6** displays signals of 2H’s of the N^2^H groups of the two thioamidate ligands in the region of 12.11–11.75 ppm and partially resolved multiplets in the region of 7.76–6.77 ppm attributed to the aromatic H’s of mqzt- and phoshpine’s phenyl groups, respectively.

### 2.2. Photophysical Properties of Complexes **1**–**6**

**Figure 4:**
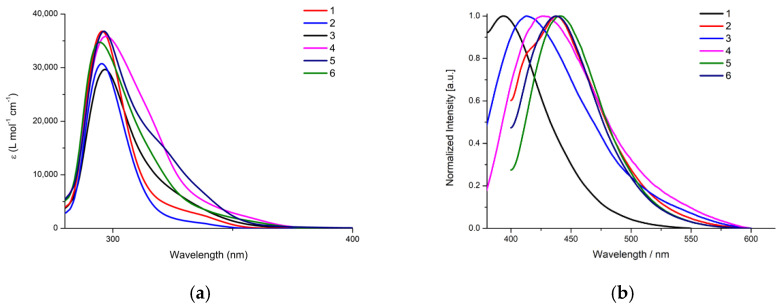
(**a**) UV-vis electronic absorption spectra of complexes **1**–**6** in DMSO solutions (5.0 × 10^−5^ mol L^−1^). (**b**) Emission spectra of complexes **1**–**6** in DMSO solutions (5.0 × 10^−5^ mol L^−1^) upon excitation at 310 nm.

The photophysical properties of complexes **1**–**6** were studied in DMSO solutions (1.0 × 10^−5^ Μ). As shown in Figure 4a, the electronic absorption spectra of the complexes display high-intensity and broad absorption bands in the UV region, with their absorption band maxima λ_max_(abs) ranging from 295 to 319 nm and the respective molar absorption coefficients ε falling between 1.5 × 10^4^ and 3.7 × 10^4^ M^−1^ cm^−1^ (Table 1). These bands are attributed to π→π* transitions of the aromatic rings of the respective ligands of the complexes. Lower-intensity and broad tail-like absorptions are observed in the lower-energy region of the absorption spectra of all complexes (in the region of 320 to 370 nm), which are attributed to intraligand charge transfer (ILCT) transitions involving the phosphine and the thioamidate ligands.

The emission spectra of complexes **1**–**6** in DMSO solutions are depicted in Figure 4b. All complexes, upon excitation at 300–360 nm, are photoluminescent. They display broad emission bands with emission band maxima λ_max_(em) falling in the blue-green region (from 394 to 442 nm) of the electromagnetic spectrum (Table 1). The emission properties of the complexes appear to arise from excited states having contributions from both the phosphine and the heterocyclic thioamide ligands. For example, comparison of the emission spectra of the isostructural mononuclear complexes **1** and **2**, displaying λ_max_(em) at 394 and 440 nm, reveals the major contribution of phosphine co-ligands (PPh_3_ and xantphos, respectively) in the emitting excited states of the complexes. In addition, the nuclearity of the complexes as well as the protonation state of the thioamide/thioamidate ligand crucially affect the emission properties of the complexes, which is clearly observed in the PPh_3_-containing complexes **1** and **3** exhibiting emission bands with λ_max_(em) at 394 and 437 nm, respectively. Overall, these preliminary findings on the emissive character of complexes **1**–**6** adequately support the potential utilization in monitoring their intracellular distribution for further investigation of their possible mechanisms of bioactivity.

The UV-vis spectra of complexes **1**–**6** were also recorded in DMSO and DMSO/ddH_2_O (5:1 *v*/*v*) mixtures over a 72 h period. No significant changes were observed in the general spectral profiles of the complexes over time compared to the initial measurements (Figures S17 and S18), indicating their good long-term stability in solution. However, a gradual increase in absorbance intensity and a slight red shift in the absorption bands were noted for all complexes. We ascribe these changes to time-dependent aggregation phenomena. To further investigate this possibility, Dynamic Light Scattering (DLS) measurements were conducted for complex **1** (as a representative example of this group of complexes) in DMSO, over a 24 h period. As shown in Figure S19, even in freshly prepared solutions aggregation occurs after 3 h, with a unimodal particle size distribution and an average particle diameter of 0.369 nm. After 24 h, the average particle size increased markedly to 1.8 μm. Therefore, these findings confirm the structural stability of the complexes in solution, which is an essential factor for reliable antibacterial and cytotoxicity assessments, but they also suggest their significant tendency towards aggregation, which may negatively impact their long-term bioactivity.

### 2.3. In Vitro Antibacterial Activity of Complexes **1**–**6**

**Figure 5:**
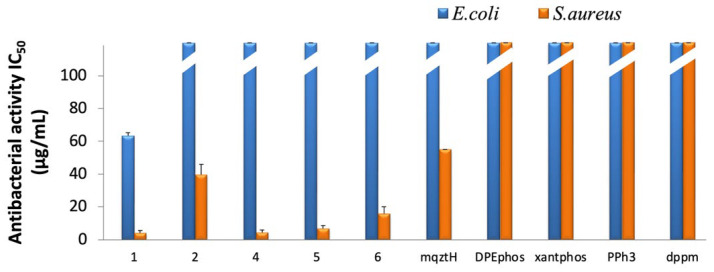
In vitro antibacterial activity of complexes **1**, **2**, **4**–**6** and their ligands mqztH, DPEphos, xantphos and PPh_3_ in free form against *E. coli* and *S. aureus* bacterial strains, expressed as half-minimum inhibitory concentration (IC_50_) values (μg mL^−1^) provided by a nonlinear curve fit-growth/sigmoidal-dose response on the experimental optical density data. Values are expressed as mean ± standard deviation (SD) of three replicate measurements (with the exception of values higher than 100 μg mL^−1^).

The in vitro antibacterial activity of the synthesized Ag(I) complexes was investigated against two bacterial strains: Gram (+) *S. aureus* and the Gram (−) *E. coli*. The assays were conducted using the broth microdilution method to determine both the half-maximal inhibitory concentrations (IC_50_) and minimal inhibitory concentrations (MIC). Due to its poor aqueous solubility, the antibacterial activity of complex **3** could not be assessed. For comparisons, the antibacterial properties of the ligands of the complexes in free form as well as the reference antibiotic ampicillin (Table S10) were also tested. The results are summarized in Figure 5 and Table S10.

Overall, complexes **1**, **2**, and **4**–**6** displayed moderate-to-high antibacterial activity against the studied bacterial strains, which was higher than that of their corresponding thioamide and phosphine ligands in free form. Moreover, all complexes appear to be more effective against *S. aureus* than against *E. coli* bacterial strain, with their IC_50_ values ranging from 4.2 to 39.7 μg mL^−1^ (4.9 to 44.2 μΜ). Notably, complexes **1**, **4**, and **5** induced significant bactericidal activity against *S. aureus* bacterial strain, with their MIC values being equal to or lower than that of ampicillin, as shown in Table S10.

Clearly, these results suggest that the complexes offer superior antibacterial efficacy, compared to conventional antibiotics, against *S. aureus* bacterial strain, while they also highlight the potential influence of their individual structural features on their bioactivity. For example, mononuclear complex **1** [Ag(mqztH)(PPh_3_)_2_Cl] demonstrated notable growth-inhibitory effects against both studied bacterial strains, with IC_50_ values of 4.2 ± 1.4 μg mL^−1^ (4.9 μΜ) and 63.3 ± 1.9 μg mL^−1^ (75 μΜ), respectively. In contrast, the structurally similar complex **2**, incorporating the bidentate chelating xantphos co-ligand, showed lower activity against *S. aureus* and no measurable activity against *E. coli* bacteria, underscoring the critical role that ligand identity and coordination mode play in modulating antibacterial performance in this class of Ag(I) complexes.

Interestingly, the structurally related complexes [Ag(dmp2SH)(PPh_3_)_2_Cl] and [Ag(dap2SH)(PPh_3_)_2_Cl], where dmp2SH = 4,6-dimethyl-pyrimidine-2-thiol and dap2SH = 4,6-diamino-pyrimidine-2-thiol, previously reported by our group, also exhibit very low IC_50_ values against both *S. aureus* and *E. coli* bacteria [24,28]. These findings reinforce the high antibacterial potential of complexes of the general formula [Ag(L^NH^S)(PPh_3_)_2_Cl], suggesting them as promising broad-spectrum antibacterial agents.

The notable antibacterial efficacy of complex **1** against Gram (−) and especially against Gram (+) bacteria may be partly attributed to the physicochemical properties and coordination behavior of its PPh_3_ co-ligands. Compared to bulkier, more rigid, and less lipophilic bidentate diphosphines (such as xantphos or DPEphos, see Table S8), PPh_3_’s smaller size and higher lipophilicity likely enhance membrane permeability, particularly in Gram (−) bacteria like *E. coli*, by promoting interactions with outer membrane porins and facilitating uptake. Additionally, the monodentate nature of PPh_3_ co-ligands may facilitate their easier dissociation from the metal centre, compared to chelating diphosphines, generating coordinatively unsaturated species with increased reactivity or affinity for membrane components, further contributing to cellular uptake. This dual effect—favourable physicochemical properties and dynamic coordination behavior—may explain the superior performance of the PPh_3_-containing complexes, such as complex **1**, against *E. coli* bacteria despite their effectiveness against *S. aureus* bacteria. Their incorporation of bidentate diphosphines, due to steric hindrance and reduced flexibility, likely limits membrane permeability and antibacterial activity.

### 2.4. Synthesis, Characterization and Properties of Hydrogels of Complex **1**

#### 2.4.1. Synthesis and Characterization

Despite the significant in vitro antibacterial activity of the herein presented Ag(I) complexes, particularly complex **1** against *S. aureus* bacteria, their high lipophilicity—although being potentially advantageous for membrane penetration—may limit their overall bioavailability and therapeutic efficacy, due to poor solubility and the presence of biological barriers in physiological conditions. Moreover, DLS measurements revealed a pronounced tendency of the complexes to self-aggregate, which could negatively affect their physical stability in solution and, consequently, diminish their bioactivity over time.

To address these limitations, complex **1**, which exhibited the highest antibacterial potency, was incorporated into an alginate matrix (**Alg**)—a biocompatible, polyanionic copolymer based on β-D-manuronic acid (M) and α-L-glucuronic acid (G) units, linearly linked to each other by 1–4 glycosidic bonds—aiming to develop **Alg**-based hydrogels [45,46]. Such an approach would offer enhanced aqueous solubility and biocompatibility of the complex, providing a “vehicle” for its potentially slower and controlled release, ultimately leading to improvement of its bioavailability and bioactivity.

**Scheme 2:**
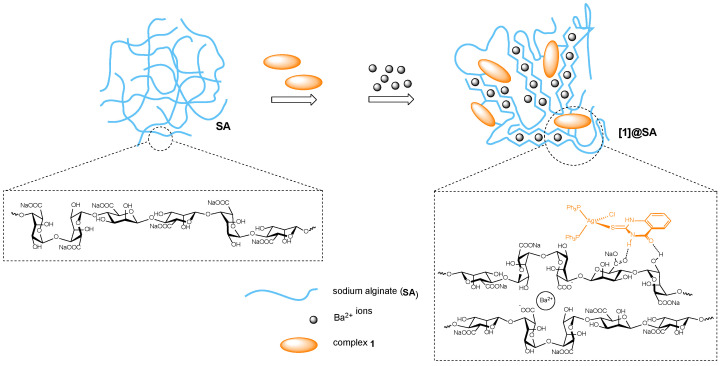
Suggested model for the intermolecular interactions developed between functional groups (NH or C=O) of the ligand mqztH of complex **1** and carboxylate groups (COO^−^) of the β-D-mannuronic acid or the OH group of the α-L-guluronic residue, respectively, of the **Alg** backbone in **[1]@BaAlg** hydrogels.

**Alg**-based hydrogel formulations of complex **1** were prepared using a straightforward ionic gelation technique following well-established protocols. Specifically, solutions of complex **1** in DMSO at three different concentrations (25, 50, and 100 μg mL^−1^) were mixed with a 2% *v*/*v* sodium alginate solution. Dropping the mixtures into 1% *v*/*v* BaCl_2_ solution resulted in the crosslinking of the alginate fibers by the Ba^2+^ ions in an egg-box-like structure and the encapsulation of complex **1**, leading to the formation of hydrogel beads **[1]@BaAlg**, designated as **[1–25]@BaAlg**, **[1–50]@BaAlg**, and **[1–100]@BaAlg**, reflecting the initial concentration of complex **1** used in each case. These were stabilized through favourable intermolecular non-covalent interactions. Specifically, hydrogen bonding interactions between functional groups on the complex, such as NH or C=O moieties from the mqztH ligand, and the carboxylate (COO^−^) or the OH groups of the **Alg** backbone are likely to play a key role (Scheme 2). These interactions not only facilitate stable incorporation of the complex into the polymeric network but may also enable controlled release of the bioactive complex.

**Figure 6:**
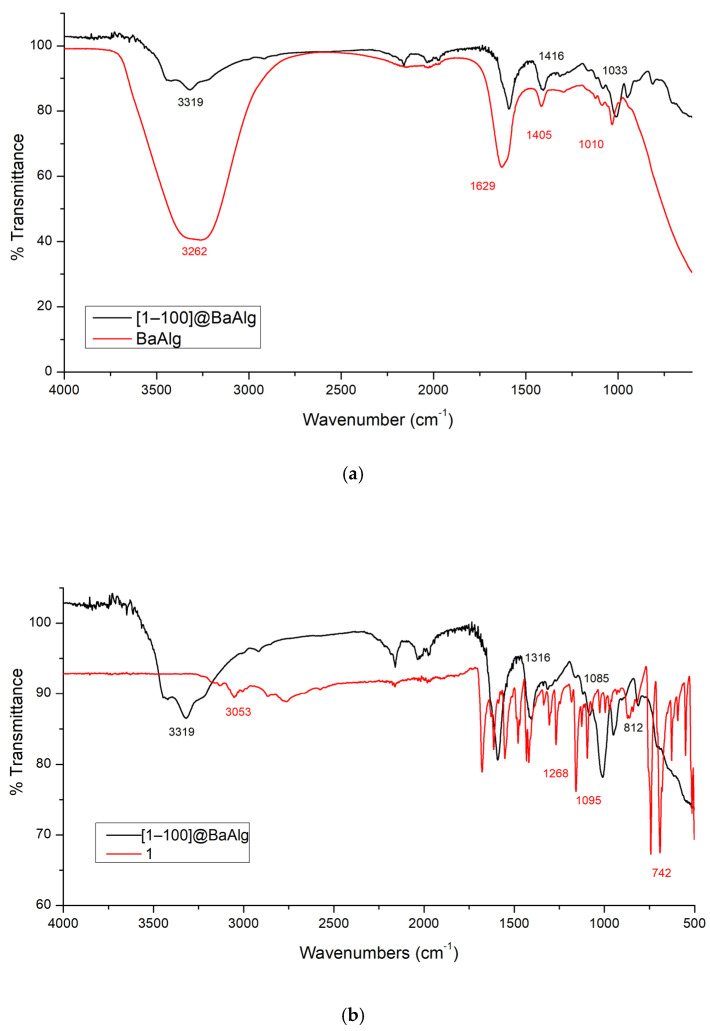
FTIR spectra of **[1–100]@BaAlg** hydrogel in comparison to (**a**) neat **BaAlg** matrix and (**b**) neat complex 1 in free form.

FTIR spectroscopy was employed to verify the successful encapsulation of complex **1** into the **BaAlg** hydrogel matrixes and to investigate potential intermolecular interactions. As shown in Figure 6a, sodium alginate exhibits characteristic absorption bands at 3262 cm^−1^ (OH stretching), 1629 and 1416 cm^−1^ (asymmetric and symmetric COO^−^ stretching), and 1032 cm^−1^ (C–O stretching). Upon incorporation of complex **1**, noticeable changes were observed, i.e., the OH band intensified and shifted to 3319 cm^−1^, while the COO^−^ bands shifted to 1591 and 1404 cm^−1^, and the C–O band shifted to 1009 cm^−1^, indicating interaction between complex **1** and the **BaAlg** matrix. Additionally, characteristic bands of complex **1** were altered upon encapsulation. The N–H stretch of the thioamide ligand at 3053 cm^−1^ shifted to 3319 cm^−1^, and the thioamide-related bands at 1552, 1268, and 1095 cm^−1^ shifted to 1558, 1316, and 1086 cm^−1^, respectively. The phosphine-associated band at 724 cm^−1^ also shifted to 812 cm^−1^. These spectral changes confirm strong interactions between the complex and the copolymer, supporting successful embedding without compromising the chemical identity of the complex.

The encapsulation efficiency (%EE) and loading capacity (%LC) of the synthesized hydrogels were calculated to assess their drug-loading performance (Table 2). All three hydrogel formulations exhibited remarkably high encapsulation efficiency, with %EE values consistently reaching 99%, which indicates that nearly all the amount of the complex was successfully entrapped within the hydrogel matrixes. Interestingly, the loading capacity increased proportionally with the initial concentration of complex **1** used. Specifically, **[1–25]@BaAlg** showed an %LC value of 0.010% and **[1–50]@BaAlg** reached an %LC value of 0.037%, while **[1–100]@BaAlg** achieved the highest %LC value of 0.050%. These results demonstrate the excellent capability and suitability of the **BaAlg** matrix for encapsulating hydrophobic Ag(I) complexes, as complex **1** presented herein, at varying concentrations.

**Figure 7:**
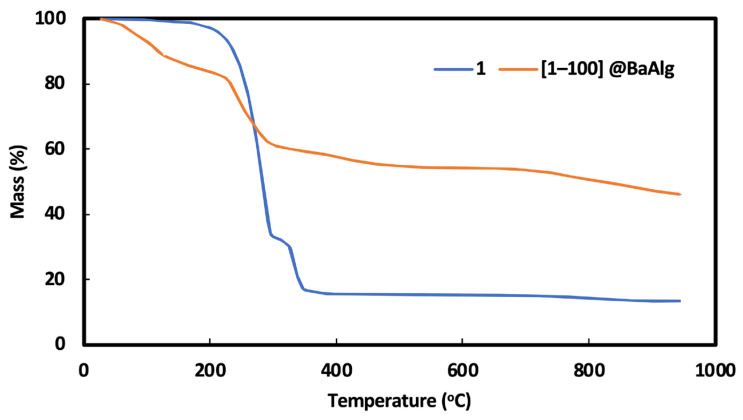
Thermogravimetric analysis curves of complex **1** (blue line) and **[1–100]@BaAlg** hydrogel (orange line).

Thermogravimetric analysis (TGA) confirmed the successful incorporation of complex **1** into the **BaAlg** matrix and the thermal stability of the resulting hydrogel formulation. As shown in Figure 7 (as well as in Figure S20a,b and Table S11), complex **1** displays two major decomposition steps: an initial mass loss of 66.42% in the temperature range of 150–308 °C, attributed to the breakdown of organic ligands, followed by a secondary loss of 17.01% from 308–400 °C, leaving 13.47% residual mass at 950 °C. **[1–100]@BaAlg** hydrogel exhibited a comparable thermal profile, with an initial 16.31% mass loss from 26 to 200 °C, due to water evaporation and possibly minor polymer degradation, followed by broader decomposition events from 200 to 500 °C (28.92%), attributed to decomposition of complex **1** and the polymer. The significantly higher residual mass of 46.06% at 950 °C indicates the presence of thermally stable Ba-containing species and confirms the structural robustness of the hydrogel matrix. These findings demonstrate not only the effective encapsulation of complex **1** in the hydrogel but also the capacity of the hydrogel to stabilize it and retain it at elevated thermal conditions.

#### 2.4.2. In Vitro Release Studies

The in vitro release kinetics of complex **1** from its **Alg**-based hydrogels were studied. Each hydrogel formulation was dispersed in PBS solution (pH 7.4) and incubated at 37 °C. Quantitative analysis of the amount of complex **1** released from each hydrogel was carried out using UV-vis spectroscopy, at time points of 24, 48, and 72 h.

**Figure 8:**
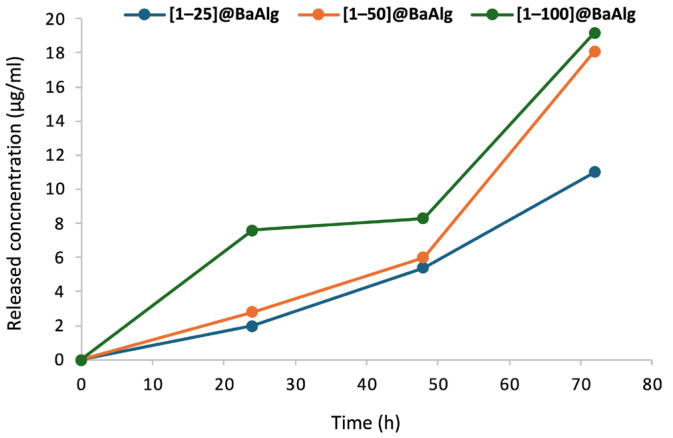
In vitro release profiles of complex **1** from the **[1–25]@BaAlg**, **[1–50]@BaAlg** and **[1–100]@BaAlg** hydrogels in PBS solution (pH 7.4), at 37 °C, over a period of 72 h.

As shown in Figure 8, **[1–25]@BaAlg** and **[1–50]@BaAlg** hydrogels exhibited similar release behaviors, releasing 2.0 and 2.8 μg mL^−1^ of complex **1**, respectively, within the first 24 h. These concentrations increased slightly after 48 h, reaching 5.4 and 6.0 μg mL^−1^. In contrast, **[1–100]@BaAlg** hydrogel showed a distinct release profile, with an initial release of 7.6 μg mL^−1^ at 24 h, followed by a minimal increase to 8.3 μg mL^−1^ in 48 h. After 72 h, all hydrogel formulations exhibited a more pronounced release, with final concentrations of 11.0, 18.1, and 19.2 μg mL^−1^ for the three hydrogel formulations, respectively. This delayed burst release pattern indicates the enhanced retention of **1** into the hydrogel network, highlights the influence of the initial complex concentration on the release dynamics and is particularly relevant for predicting the behavior of complex **1** in aqueous environments following encapsulation. These findings also provide a valuable basis for evaluating its sustained bioactivity in hydrogel form.

### 2.5. In Vitro Antibacterial Activity Studies of Hydrogels of Complex **1**

#### 2.5.1. In Vitro Antibacterial Activity Studies

**Figure 9:**
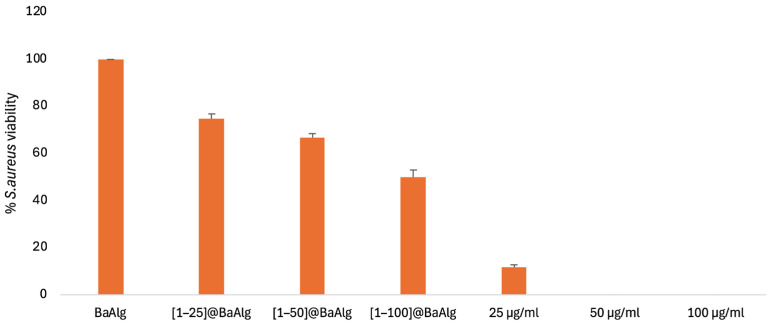
In vitro viability (%) of *S. aureus* bacterial strain after 24 h incubation with complex **1** in free form at concentrations of 25, 50 and 100 μg mL^−1^, as well as **BaAlg**, and **[1–25]@BaAlg**, **[1–50]@BaAlg** and **[1–100]@BaAlg** hydrogels.

The in vitro antibacterial activity of the **[1–25]@BaAlg**, **[1–50]@BaAlg**, and **[1–100]@BaAlg** hydrogels was evaluated against the *S. aureus* bacterial strain. Bacteria were exposed to the three **Alg**-based hydrogel formulations, neat **BaAlg** hydrogel and three solutions of complex **1** at concentrations of 25, 50, and 100 μg mL^−1^, over a period of 24 h. The results of the study are summarized in Table 3 and Figure 9.

The hydrogel formulations exhibited dose-dependent antibacterial effects, inhibiting *S. aureus* bacterial growth by ca. 25, 33, and 50%, respectively. In contrast, neat **BaAlg** hydrogel had no adverse impact on bacterial growth, confirming that the antibacterial activity originated from the encapsulated complex. These findings, when considered alongside with the release profile of complex **1** from its **Alg**-based hydrogels, indicate that complex **1** retains its antibacterial properties upon incorporation into the **BaAlg** matrix. Specifically, within 24 h, the actual released concentrations of complex **1** from the **[1–25]@BaAlg**, **[1–50]@BaAlg**, and **[1–100]@BaAlg** hydrogels were 2.0, 2.8, and 7.6 μg mL^−1^, respectively. These effective concentrations, being lower by an order of magnitude compared complex **1** in its free form, induce a moderate antibacterial effect, with the hydrogel with the higher loading achieving a 50% reduction in bacterial viability. Specifically, the released concentration of 7.6 μg mL^−1^ of complex **1** from the **[1–100]@BaAlg** hydrogel slightly exceeds the IC_50_ of complex **1** in its free form (4.2 μg mL^−1^) and, therefore, the encapsulated form of the complex appears to be slightly less potent (possibly due to slower uptake or even partial hydrolysis). However, the significantly lower cytotoxicity observed of all hydrogels suggests enhanced therapeutic indexes, making them more suitable for biomedical applications. A comparable trend has been reported for Ag(I) sulfadiazine embedded in alginate/chitosan scaffolds, which also demonstrated moderate antibacterial activity against *S. aureus* bacterial strain. This controlled and sustained release approach is advantageous in the treatment of chronically infected wounds, as it reduces the risk of side effects often associated with high single-dose therapies. Therefore, the controlled delivery of complex **1** via **BaAlg** hydrogels presents a promising strategy for safe and effective antibacterial treatment.

#### 2.5.2. In Vitro Cytotoxicity Studies

**Figure 10:**
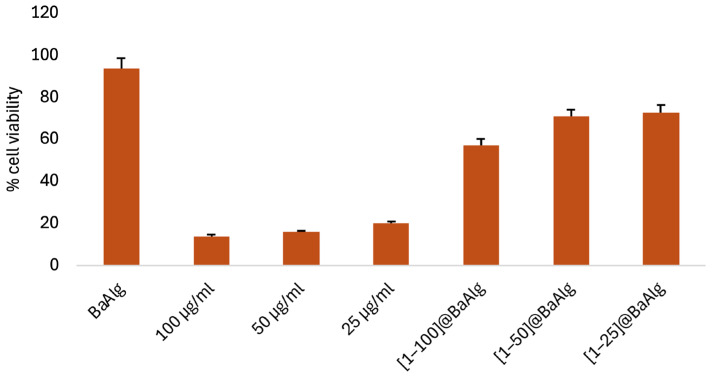
In vitro viability (%) of L929 normal cell line after 48 h incubation with complex **1** in free form at concentrations of 25, 50 and 100 μg mL^−1^, as well as **BaAlg**, and **[1–25]@BaAlg**, **[1–50]@BaAlg** and **[1–100]@BaAlg** hydrogels.

To evaluate the potential of the synthesized hydrogels to act as reduced-cytotoxicity antibacterial agents, in vitro cytotoxicity studies were performed against the L929 normal fibroblast cell line. Cells were exposed to the **[1–25]@BaAlg**, **[1–50]@BaAlg**, and **[1–100]@BaAlg** hydrogels, neat **BaAlg** hydrogel and three solutions of complex **1** at concentrations of 25, 50, and 100 μg mL^−1^. Following a 48 h incubation period, the cytotoxic effects of all agents were assessed and compared to determine their impact on normal cell viability. The results of the study are summarized in Table 4 and Figure 10.

Complex **1** in its free form exhibited a strong cytotoxic effect, resulting in cell viabilities of only 14.1, 16.1, and 20.2% at concentrations of 25, 50, and 100 μg mL^−1^, respectively. In contrast, hydrogels of complex **1**, as well as the neat **BaAlg** hydrogel, did not significantly affect cell viability. Specifically, treatment of the cells with **[1–25]@BaAlg**, **[1–50]@BaAlg** and **[1–100]@BaAlg** hydrogels led to only a 25–40% reduction in cell viability, indicating that encapsulation of complex **1** within the **BaAlg** matrix substantially mitigates its cytotoxicity against the studied normal cells.

The findings presented herein are encouraging for the potential utilization of these **Alg**-based hydrogel formulations of complex **1** in biomedical applications. Notably, when compared to literature data on similar systems, they demonstrate superior biocompatibility. For instance, Ag(I) sulfadiazine embedded in 3D-printed **Alg**/chitosan scaffolds showed improved biocompatibility over 24 h with respect to the free drug [47]. In comparison, **[1]@BaAlg** hydrogels exhibited ca. 2-fold lower cytotoxicity than Ag(I) sulfadiazine-containing hydrogels and maintained cell viability over a longer exposure period (48 h). The observed differences in the cytotoxicity between the hydrogel formulations of complex **1** and Ag(I) sulfadiazine likely stem from variations in their release profiles and mechanisms of bioactivity [48].

### 2.6. Mechanistic Investigations

To gain insight into potential mechanisms underlying the observed in vitro bioactivity of the herein presented Ag(I) complexes, a series of mechanistic studies was carried out, focusing on complex [AgCl(mqztH)(PPh_3_)_2_] (**1**), which exhibited the strongest antibacterial activity against both Gram (+) and Gram (−) bacterial strains.

#### 2.6.1. In Vitro Effect of Complex **1** on L929 Normal Cell Line

**Figure 11:**
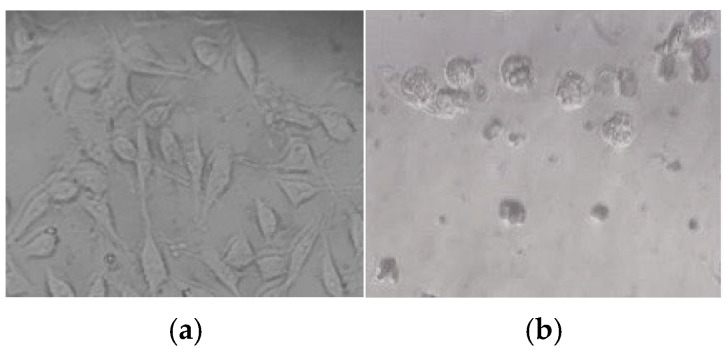
Optical microscopy images (scale bar 100 μm) of (**a**) untreated L929 normal cell line and (**b**) L929 normal cell line treated with a 25 μg mL^−1^ solution of complex **1** for 48 h.

The morphological changes that L929 normal eukaryotic cells undergo after their in vitro treatment with solutions of complex **1** were examined by optical microscopy. The changes in treated cells may provide useful insights into the mechanisms of cell death. Cells were treated with solutions of complex **1** at concentrations of 25, 50, and 100 μg mL^−1^ for 48 h. Figure 11b shows that treatment of the cells with the 25 μg mL^−1^ solution of complex **1** resulted in notable morphological changes, such as their detachment and adoption of a spherical shape, features commonly associated with cytotoxic effects. At higher concentrations (50 and 100 μg mL^−1^), the extent of cytotoxicity increased, resulting in significant cell detachment and extensive structural disruption. The damage was so severe that clear and interpretable microscopy images were not possible to be obtained. This suggests that the cells were not only morphologically altered, but they also underwent lysis or irreparably damaged. Such extensive damage is indicative of disruption of the cell membrane integrity, a key characteristic of cytotoxicity and is typical of necrotic cell death or later phases of apoptosis.

#### 2.6.2. Redox Behavior of Complex **1**

**Figure 12:**
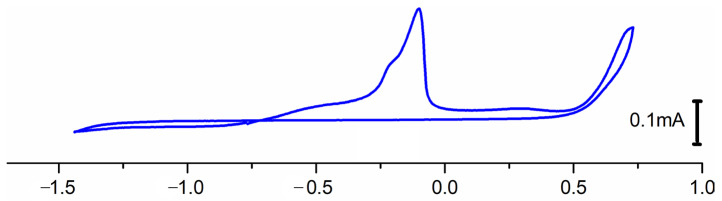
Cyclic voltammogram of complex **1** in CH_3_CN.

The redox behavior of complex [AgCl(mqztH)(PPh_3_)_2_] (**1**) was examined by cyclic voltammetry (CV). CV measurements were conducted in CH_3_CN, revealing distinct oxidation and reduction events (Figure 12). When the scan is initiated at ca. +0.1 V (rest potential) toward positive potentials, a single oxidation wave is observed with an onset of ca. +0.5 V and a peak at ca. +0.8 V, with no corresponding return wave, indicating an irreversible oxidation process. Considering that the thioamide ligand itself also undergoes an irreversible oxidation in this potential region (Figure S21), this process is most-likely attributed to a ligand-centered irreversible oxidation (possibly, forming a nitrogen-centered radical cation on the ligand). Scans toward negative potentials (initiated at the rest potential) reveal a cathodic event which starts appearing at ca. −0.75 V and shows curve crossing, which is attributed to a metal-centered reduction of the complex and deposition of metallic Ag(0). Specifically, upon reduction of the complex, ligand dissociation occurs—liberating the thioamide mqztH, Cl^−^ ion, and PPh_3_ molecules—which ultimately results in the formation of metallic Ag(0) deposited on the working electrode surface: [AgCl(mqztH)(PPh_3_)_2_] + e^−^ → [AgCl(mqztH)(PPh_3_)_2_]^−^ → Ag(0) + Cl^−^ + mqztH + 2PPh_3_. Analogous non-linear CV behavior (with curve crossing), associated with nucleation and growth of metallic Ag(0) and alteration of the electrode surface, has been reported for other Ag(I) complexes [49]. On the return scan (anodic scan), a sharp and high-intensity oxidation peak appears, initiating at ca. –0.2 V and reaching a peak at ca. 0.1 V. This feature is absent in scans initiated anodically, while it also varies in intensity across successive cycles and scan rates (Figures S22 and S23), and, therefore, it is attributed to a surface-confined anodic stripping of deposited Ag(0) back to Ag^+^: Ag(0) → Ag^+^ + e^−^. In addition, a broad oxidation signal is observed in the range of ca. −0.6 to −0.25 V, which is assigned to the oxidation of Cl^−^ ions released after the reduction/decomposition of the complex—a diffusion-controlled process that depends on the scan rate [49,50]. Following this oxidation process of the reduction cycle, an irreversible oxidation appears, arising from the thioamide-centered oxidation, as discussed above. Overall, the CV behavior of complex **1** is dominated by irreversible electron transfers, electrode surface processes, and ligand dissociation dynamics, typical for Ag(I) complexes under reductive conditions.

The redox activity of complex **1** occurs at potentials falling within or near the biologically accessible electrochemical window (typically, between −0.9 and 0.0 V vs. Fc^+^/Fc in aqueous solutions at pH 7.0) [51], which suggests it has the potential for causing antibacterial effects through redox-mediated mechanisms. After its interaction with the membrane of the bacterial cell or cellular internalization, reductants, such as NADH or enzymes like GR and TrxR, can reduce the Ag(I) center to metallic Ag(0), a transformation that lies within the biological redox window. Metallic Ag(0) so formed has the potential to interact with thiol groups of protein factors involved in cellular redox equilibria and metabolic processes and, therefore, cause cell damage. Metallic Ag(0) can also get oxidized by molecular oxygen, forming Ag(I) ions and reactive oxygen species (ROS), such as hydrogen peroxide, H_2_O_2_, (E° ≈ +0.45 V) and superoxide, O_2_^•−^. This kind of redox cycle of Ag(I)/Ag(0) sustains the level of oxidative stress inside the bacterial cell, thereby enhancing the antibacterial efficacy of the complex. In parallel, the irreversible mqztH-centered oxidation of the complex at more positive potentials (onset ca. +0.5 V, peak ca. +0.8 V vs. Fc^+^/Fc), might also play a role in cellular redox perturbation. Despite the fact that this oxidation falls outside the biological redox window, it lies near the potential for the formation of H_2_O_2_ from molecular oxygen, which means that under oxidative stress conditions, the complex might be able to generate ROS. This oxidative conversion of the thioamide ligand may mimic endogenous oxidative damage and may also interfere with redox-active enzymes. Overall, these redox processes, i.e., cycling of Ag(I)/Ag(0), production of ROS, and ligand-centered oxidative reactivity, form the basis of a multi-faceted antibacterial mechanism involving disruption of redox homeostasis and destabilization of intracellular redox buffers, such as NAD^+^/NADH and GSH/GSSG [52]. These modes of action are in line with accepted cytotoxic mechanisms of redox-active Ag(I)-based antimicrobial agents, and suggest that the redox capacity of complex **1** plays an integral role in its bioactivity [53].

#### 2.6.3. Inhibition of Bacterial GR and TrxR by Complex **1**

GR and TrxR are essential enzymes for the retention of systemic redox homeostasis of bacteria cells and thus are considered as antimicrobial drug targets for the effective inhibition of bacteria growth [54,55]. Based on the results of the cyclic voltammetry study, as part of the mechanistic studies, the activity of complex **1** was tested against both GR and TrxR pure enzymes isolated from *E. coli* bacteria. The bioactivity of the complex was compared to AgNO_3_ which was used as reference compound and found to demonstrate strong inhibition of both GR and TrxR. The inhibition of *E. coli* TrxR and GR was determined as previously published [56], and the results are shown in Table 5. The activity against the latter enzyme is rather non-selective, with IC_50_ values found in a narrow nanomolar range (0.031 µM of **1** and 0.061 µM of AgNO_3_, respectively). In contrast, the activity against bacterial TrxR varies drastically between **1** and AgNO_3_. While **1** appears to be a potent inhibitor of the enzyme, it still shows much weaker effects than the free silver ion source (AgNO_3_). For comparison, Ag(I) complexes bearing either N-heterocyclic carbenes or antibiotics as ligands displayed promising inhibitory activity which is similar to **1** and generally better than the common antibiotics in free form, indicating the importance of the synergistic effect of Ag(I) and its ligands on bacterial strains [57,58]. This also suggests that inhibition of both thioredoxin and glutathione reductases could be a possible mechanism for the bioactivity of the herein presented Ag(I) compounds and critical for their high efficacy on bacteria.

## 3. Conclusions

Six mononuclear Ag(I) complexes with mqztH as ligand, in combination with various phosphine co-ligands, were synthesized and assessed for their in vitro antibacterial activity. The complexes demonstrated significant bacteriostatic activity against the *S. aureus* bacterial strain (IC_50_ = 4.9–13 μΜ), particularly those having PPh_3_ as co-ligand, likely due to its flexible coordination behavior and favorable physicochemical features. Among them, broad-spectrum efficacy against Gram (+) and Gram (−) bacterial strains was shown by [AgCl(mqztH)(PPh_3_)_2_] (**1**). To improve its bioactivity, complex **1** was encapsulated in barium alginate matrices to produce efficient and biocompatible hydrogel-based drug delivery systems **[1]@BaAlg**, an innovative approach which is scarcely applied for controlled release of Ag(I) complexes. In vitro antibacterial tests confirmed that complex **1** retained its bactericidal activity after its encapsulation into the hydrogels, with its inhibitory concentrations have been ~10-fold lower (2.0 μg mL^−1^) compared to the free complex (25 μg mL^−1^). Cytotoxicity tests on the L929 normal cell line demonstrated good biocompatibility, preserving about 70% cell viability. Mechanistic studies confirmed the redox-active nature of complex **1**, capable of undergoing redox processes through both metal- and ligand-centered pathways, and its potential to inhibit the function of bacterial GR and TrxR at low concentrations, suggesting that disrupting bacterial redox balance may be a relevant mechanism of its bioactivity. Overall, these results highlight the potential of Ag(I) complexes with the particular quinazolinone thioamide ligand as efficient redox-active antibacterial agents and, together with their **Alg**-based hydrogel formulations as effective drug-delivery platforms, suggest a promising direction to antibacterial drug design and development.

## Figures and Tables

**Table 1:** Photophysical characteristics of complexes **1**–**6**: absorption band maxima λ_max_(abs) (nm), molar absorption coefficients ε (M^−1^ cm^−1^) and emission band maxima λ_max_(em) (nm) in DMSO solutions (5.0 × 10^−5^ mol L^−1^) upon excitation at 310 nm.

	λ_max_(abs)/nm (ε/M^−1^ cm^−1^)	λ_max_(em)/nm
[AgCl(mqztH)(PPh_3_)_2_] (**1**)	295 (37,000), 337 (2500)	394
[AgCl(mqztH)(xantphos)] (**2**)	295 (31,000), 337 (1000)	440
[Ag(mqzt)(PPh_3_)]_2_ (**3**)	297 (30,000)	437
[Ag(mqzt)(dppm)]_2_ (**4**)	297 (35,000), 317 (20,000), 356 (2000)	428
[Ag(mqzt)(DPEPhos)]_2_ (**5**)	296 (37,000), 319 (16,000), 340 (6000)	442
[Ag(mqzt)(xantphos)]_2_ (**6**)	295 (35,000), 356 (1500)	438

**Table 2:** Loading capacity (%) and encapsulation efficiency (%) values for **[1–25]@BaAlg**, **[1–50]@BaAlg** and **[1–100]@BaAlg** hydrogels.

	%LC	%EE
**[1–25]@BaAlg**	0.010	99
**[1–50]@BaAlg**	0.037	99
**[1–100]@BaAlg**	0.050	99

**Table 3:** In vitro antibacterial activity of complex **1** in its free form at concentrations of 25, 50 and 100 μg mL^−1^, and **[1–25]@BaAlg**, **[1–50]@BaAlg**, **[1–100]@BaAlg** hydrogels, against *S. aureus* bacteria after 24 h incubation. Actual concentrations of complex **1** for the **Alg**-based hydrogels are reported those released after 24 h.

	Actual Concentration of Complex 1 (μg mL^−1^)	% Viability of *S. aureus* Bacteria
**1**	25 μg mL^−1^	12
	50 μg mL^−1^	0
	100 μg mL^−1^	0
**[1–25]@BaAlg**	2.0	75
**[1–50]@BaAlg**	2.8	67
**[1–100]@BaAlg**	7.6	50

**Table 4:** In vitro cytotoxicity of complex **1** in its free form at concentrations of 25, 50 and 100 μg mL^−1^, and **[1–25]@BaAlg**, **[1–50]@BaAlg**, **[1–100]@BaAlg** hydrogels, against L929 normal fibroblast cell line after 48 h incubation. Actual concentrations of complex **1** for the **Alg**-based hydrogels are reported those released after 48 h.

	Actual Concentration of Complex 1 (μg mL^−1^)	% Viability of L929 Normal Cells
**1**	25	20
	50	16
	100	14
**[1–25]@BaAlg**	5.4	75
**[1–50]@BaAlg**	6.0	71
**[1–100]@BaAlg**	8.3	60

**Table 5:** Inhibition of *E. coli* GR and TrxR by complex **1** and AgNO_3_ expressed as IC_50_ values (μM). Values in parentheses represent the standard errors obtained from three independent experiments.

	*E. coli* GR	*E. coli* TrxR
	IC_50_ (μΜ)	
[AgCl(mqztH)(PPh_3_)_2_] (**1**)	0.065 (±0.013)	0.215 (±0.043)
AgNO_3_	0.031 (±0.001)	0.061 (±0.008)

## Data Availability

The original contributions presented in this study are included in the article/Supplementary Materials. Further inquiries can be directed to the corresponding authors.

## Supplementary Materials

The following supporting Information can be downloaded at: https://www.mdpi.com/article/10.3390/molecules30204071/s1, Experimental-Sections: S1.1 General procedures and chemicals; S1.2 Syntheses complexes **1**–**6**; S1.3 Synthesis and characterization of **[1]@BaAlg** hydrogels, S1.4 Instrumentation; S1.5 X-ray crystal structure determinations; S1.6 In vitro antibacterial activity studies; S1.7 Mechanistic investigations. Results-Sections: S2.1 Single-crystal X-ray structural analysis (Figures S1–S10 and Tables S1–S8—X-ray crystal structures of complexes **1**–**6**); S2.2. FTIR spectroscopy (Table S9—FTIR spectra of complexes **1**–**6**); S2.3 ^1^H NMR spectroscopy (Figures S11–S16 ^1^H NMR spectra of complexes **1**–**6**); S2.4 Stability studies in solution (Figures S17 and S18—UV-vis spectra of complexes **1**–**6** recorded in solution over time); S2.5 Dynamic Light Scattering studies (Figure S19—Hydrodynamic average particle diameter distribution profiles of complex **1** in solution by DLS measurements recorded over time; S2.6 In vitro antibacterial activity studies (Table S10—Antibacterial activity studies of complexes **1**–**6** expressed as IC_50_ values); S2.7 Thermogravimetric analysis (Figure S20 and Table S11—Thermogravimetric analysis results of complex **1** and **[1]@BaAlg** hydrogel. 2.8 Electrochemical studies (Cyclic voltammograms of thioamide ligand and complex **1**, Figures S21–S23). References [59,60,61,62,63,64,65,66] are cited in the Supplementary Materials. Crystallographic information files (cif) for complexes **1**–**6**—Accession Codes: CCDC 2474674, 2474675, 2474676, 2474677, 2474678, 2474679. These data can be obtained free of charge via https://www.ccdc.cam.ac.uk/structures/, or from the Cambridge Crystallographic Data Centre, 12 Union Road, Cambridge CB2 1EZ, UK; fax: (+44) 1223-336-033; or email: deposit@ccdc.cam.ac.uk.
