# Supplementary material for: Redox-Active Quinazolinone Thioamide Ag(I) Complexes with Potent Antibacterial Activity: Mechanistic Insights and Hydrogel-Enhanced Efficacy
Source: Molecules. 2025 Oct 13;30(20):4071. doi: 10.3390/molecules30204071 (PMC12566157; doi:10.3390/molecules30204071)
Supplement: Supplementary file 1 [file molecules-30-04071-s001.zip › molecules-3872931-supplementary.pdf]

Supporting Information

Redox-Active Quinazolinone Thioamide Ag(I) Complexes with Potent Antibacterial Activity: Mechanistic Insights and Hydrogel-Enhanced Efficacy

Eleni Ioanna Tzaferi [1,†], Despoina Varna [1,†], Igor V. Esarev [2], Konstantina Kavaratzi [1], Antonios G. Hatzidimitriou [1], Rigini Papi [3], Ingo Ott [2,*] and Panagiotis A. Angaridis [1,*]

[1] Laboratory of Inorganic Chemistry, Department of Chemistry, Aristotle University of Thessaloniki, 54124 Thessaloniki, Greece
[2] Institute of Medicinal and Pharmaceutical Chemistry, Technische Universität Braunschweig, 38106 Braunschweig, Germany
[3] Laboratory of Biochemistry, Department of Chemistry, Aristotle University of Thessaloniki, 54124 Thessaloniki, Greece
* Correspondence: ingo.ott@tu-bs.de (I.O.); panosangaridis@chem.auth.gr (P.A.A.)
† These authors contributed equally to this work.

TABLE OF CONTENTS

S1 EXPERIMENTAL SECTION 3

S1.1 General procedures and chemicals 3

S1.2 Synthesis of complexes 1-6 3

S1.2.1 [AgCl(mqztH)(PPh_3)$_2$] (1) 3

S1.2.2 [AgCl(mqztH)(xantphos)] (2) 3

S1.2.3 [Ag(mqzt)(PPh_3)]$_2$ (3) 4

S1.2.4 [Ag(mqzt)(DPEPhos)]$_2$ (4) 4

S1.2.5 [Ag(mqzt)(dppm)]$_2$ (5) 5

S1.2.6 [Ag(mqzt)(xantphos)]$_2$ (6) 5

S1.3 Synthesis and characterization of [1–25]@BaAlg, [1–50]@BaAlg and [1–25]@BaAlg hydrogels 6

S1.3.1 Synthesis of hydrogels 6

S1.3.2 Loading capacity and encapsulation efficiency of hydrogels 6

S1.4 Instrumentation 6

S1.5 Single-crystal X-ray diffraction analysis and calculation of steric effect of ligands 7

S1.6 In vitro antibacterial activity studies 8

S1.6.1 Broth microdilution assay 8

S1.7 Mechanistic investigations 8

S1.7.1 In vitro Glutathione reductase (GR) and Thioredoxin reductase (TrxR) inhibition assay 8

S2 RESULTS 9

S2.1 Single crystal X-ray diffraction analysis 9

S2.2 FTIR spectroscopy 21

S2.3 ^{1}H NMR spectroscopy 22

S2.4 Stability studies 25

S2.5 Dynamic Light Scattering studies 27

S2.6 In vitro antibacterial activity studies 28

S2.7 Thermogravimetric analysis 29

S2.8 Electrochemical studies 30

S1 EXPERIMENTAL SECTION

S1.1 General procedures and chemicals

All manipulations were carried out under atmospheric conditions, unless otherwise mentioned. Solvents were purified according to established methods and allowed to stand over molecular sieves for 24 h. Silver(I) starting materials, i.e., silver nitrate ($AgNO_3$), 2-mercapto-4(3H)-quinazolinone (mqztH), thriphenylphosphine (PPh_3), 4,5-bis(diphenylphosphano)-9,9-dimethylxanthene (xantphos), bis[(2-diphenylphosphino)phenyl]ether (DPEphos) and bis(diphenylphosphino)methane (dppm) were obtained from commercial sources and used without any further purification. For the cytotoxicity studies, 3-(4,5-dimethylthiazol-2-yl)-2,5-diphenyltetrazolium bromide (MTT) and dimethyl sulfoxide (DMSO) were purchased from Sigma-Aldrich (St. Louis, MO, USA). Dulbecco's modified Eagle's medium (DMEM), fetal bovine serum (FBS) and penicillin/streptomycin (10,000 U/mL) were purchased from Gibco (Life Technologies, NY, USA).

S1.2 Synthesis of complexes 1-6

S1.2.1 [AgCl(mqztH)(PPh$_3$)$_2$] (1)

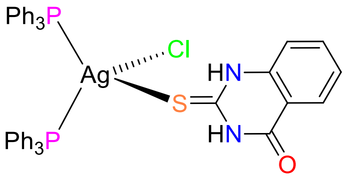

To a suspension of AgCl (0.043 g, 0.3 mmol) in 30 mL of CH_3CN, PPh_3 (0.157 g, 0.6 mmol) was added in small portions, and the resulting mixture was stirred for 24 h at 50 °C and in the dark. A portion of mqztH (0.054 g, 0.3 mmol) was added, the reaction mixture was stirred for 2 h and then was filtered. The filtrate was set aside in dark to evaporate slowly. Large white crystals of **1** were grown over a period of 8 days, which were collected. Crystals of **1** were dried under vacuum to afford the solvent-free product. Yield: 0.027 g (11%) Anal. Calcd for [$C_{44}H_{36}AgClN_2OP_2S$]: % C, 62.46; H, 4.29; N, 3.31. Found: % C, 62.56; H, 4.39; N, 3.41. FTIR (KBr, cm^{-1}): 512 (s), 533 (m), 552 (m), 582 (m), 693 (s), 745 (s), 790 (m), 879 (m), 959 (w), 998 (w), 1023 (w), 1097 (w), 1236 (m), 1297 (m), 1406 (m), 1518 (m), 1632 (m), 2976 (m), 3051 (m). ^{1}H NMR (500 MHz, DMSO-d_6): δ (ppm): 8.99 (s, 1H, N^{1}H), 8.06-8.05 (d, 1H, N^{2}H), 7.75-7.74 (d, 1H, mqztH), 7.67-7.65 (m, 3H, mqztH), 7.44-7.41 (m, 12H, m-H, PPh_3), 7.32-7.31 (m, 6H p-H, PPh_3), 7.26-7.23 (m, 12H, m-H, PPh_3). UV-Vis (CH_3CN), λ_{max}/nm (logε): 295 (4.56), 337 (3.39)

S1.2.2 [AgCl(mqztH)(xantphos)] (2)

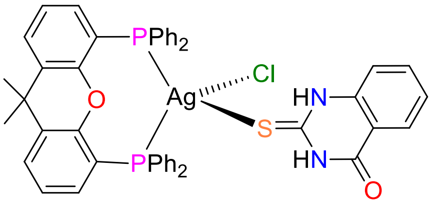

AgCl (0.043 g, 0.3 mmol) was suspended in 30 mL of CH_3CN, xantphos (0.174 g, 0.3 mmol) was added in small portions, and the resulting mixture was stirred at 50 °C for 6 h in the dark. Then, mqztH (0.054 g, 0.3 mmol) was added and the reaction mixture was stirred for 2 h. After filtration of the reaction mixture, a small amount of a white solid was removed, and the filtrate was set aside to evaporate slowly. Over a period of 20 days, large white crystals of **2** were obtained. Crystals of **2** were dried under vacuum to afford the solvent-free product. Yield 0.023g (9%). Anal. Calcd for [$C_{47}H_{38}AgClN_2O_2P_2S$]: % C, 62.71; H, 4.26; N, 3.11. Found: % C, 60.41; H, 4.06; N, 3.09. FTIR (KBr, cm^{-1}):

510 (s), 532 (m), 547 (w), 590 (m), 695 (s), 740 (s), 749 (s), 771 (s), 917 (w), 965 (m), 999 (m), 1024 (m), 1058 (m), 1100 (m), 1226 (s), 1354 (m), 1404 (s), 1435 (s), 1603 (w), 2960 (w), 3054 (w). ^{1}H NMR (500 MHz, DMSO-d_6): δ (ppm): 12.75 (s, 1H, N^{1}H), 12.47 (s, 1H, N^{2}H), 7.92-7.90 (dd, 1H, mqztH), 7.72-7.70 (m, 3H, mqztH), 7.40-7.37 (m, 8H, m-H, phenyl), 7.36-7.35 (m, 4H, p-H, phenyl), 7.32-7.29 (m, 8H, o-H, phenyl), 7.23-7.20 (t, 2H, xantphos backbone), 6.92-6.90 (t, 1H, xantphos backbone), 6.78-6.77 (d, 1H, xantphos backbone), 6.58-6.56 (m, 2H, xantphos backbone), 1.56 (s, 6H, CH_3). UV-Vis (DMSO), λ/nm (logε): 295 (4.49), 337 (3.00)

S1.2.3 [Ag(mqzt)(PPh$_3$)]$_2$ (3)

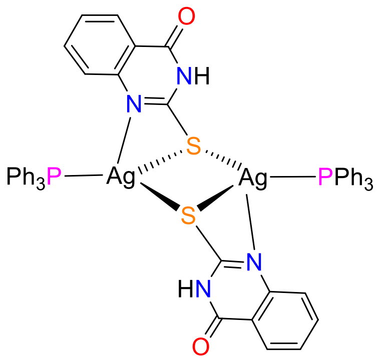

A portion of $AgNO_3$ (0.051 g, 0.3 mmol) was dissolved in 10 mL of CH_2Cl_2, then PPh_3 (0.157 g, 0.6 mmol) was added, and the resulting solution was stirred at room temperature for 45 min in the dark. A solution of - K^+(mqzt)$^-$ in 10 mL of CH_2Cl_2, obtained by deprotonation of the corresponding amount of mqztH (0.054 g, 0.3 mmol) with 1.5 mL of 0.2 M methanolic solution of KOH, was added dropwise and the reaction mixture was further stirred for 1 h at 60 °C. The resulting suspension was allowed to cool at room temperature, then filtered in order to remove a small amount of white solid and layered by Et_2O. Large white crystals of **3** were formed over a period of 10 days, which were collected. Crystals of **3** were dried under vacuum to afford the solvent-free product. Yield (Based on Ag): 0.035 g (21 %). Anal. Calcd for [$C_{52}H_{40}Ag_2N_4O_2P_2S_2$]: % C, 57.05; H, 3.68; N, 5.12. Found: % C, 57.15; H, 3.78; N, 5.22. FTIR (KBr, cm^{-1}): 511 (s), 546 (s), 577 (m), 650 (w), 693 (s), 721 (m), 745 (s), 761 (s), 791 (m), 811 (m), 852 (m), 916 (m), 1000 (m), 1098 (m), 1239 (s), 1386 (m), 1406 (m), 1506 (s) 1568 (m), 1601 (m), 1677 (m), 2980 (w), 3050 (w). ^{1}H NMR (500 MHz, DMSO-d$_6$): δ (ppm): 12.05 (s, 2H, N^{2}H), 7.59-7.54 (m, 2H, mqzt), 7.43-7.41(m, 2H, mqzt), 7.40-7.39(m, 12H, o-H), 7.36-7.34 (m, 6H, p-H), 7.31-7.28 (m, 12H, m-H), 7.13-7.06 (m, 4H, mqzt). UV-Vis (DMSO), λ_{max}/nm (ε/M^{-1} cm^{-1}): 297 (4.48).

S1.2.4 [Ag(mqzt)(DPEPhos)]$_2$ (4)

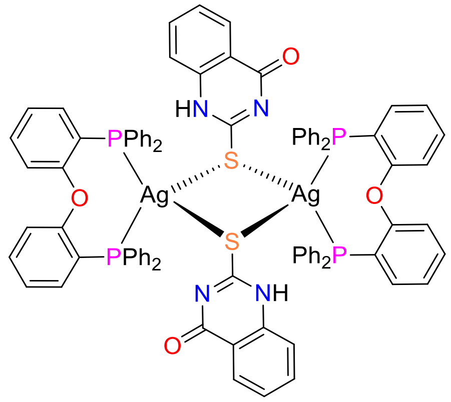

$AgNO_3$ (0.051 g, 0.3 mmol) was dissolved in 20 mL of CH_2Cl_2. Then DPEphos (0.162 g, 0.3 mmol) was added in small portions, and the resulting mixture was stirred at 50 °C for 45 min in the dark. A solution of K^+(mqzt)$^-$, obtained by the addition of 1.5 mL of 0.2 M methanolic solution of KOH into a solution of mqztH (0.054 g, 0.3 mmol) in 10 mL of CH_2Cl_2, was added and the reaction mixture was further stirred in ambient conditions for 2 h. After filtration, the filtrate was layered with Et_2O. White crystals of **4** were grown over a period of 20 days, which were collected. Crystals of **4** were dried under vacuum to afford the solvent-free product. Yield: 0.198 g (73%). Anal. Calcd for [$C_{88}H_{66}Ag_2N_4O_4P_4S_2$]: % C, 64.16; H, 4.04; N, 3.40. Found: % C, 64.56; H, 4.44; N, 3.74. FTIR (KBr, cm^{-1}): 518.7 (s), 531.6 (m),

577.7 (m), 622.05 (m), 693.06 (s), 747.47 (s), 803.79 (w), 964.64 (m), 1024.14 (m), 1069.28 (m), 1093.63 (m), 1217.63 (m), 1259.04 (w), 1351.68 (w), 1434.01 (s), 1561.04 (w), 3048.71 (w). ^{1}H NMR (500 MHz, DMSO-d_6): δ (ppm) 11.74 (s, 2H, mqzt, N^2H), 7.81 (s, 2H, mqzt), 7.42-7.38 (m, 16H, o-H, phenyl), 7.39-7.33 (m, 16H, m-H, phenyl), 7.28-7.29 (m, 4H, DPEphos, backbone), 7.07-7.04 (t, 8H, p-H, phenyl), 6.99 (s, 4H, DPEphos, backbone), 6.86 (s, 6H, mqzt), 6.77 (s, 4H, DPEphos, backbone), 6.69 (s, 4H, DPEphos, backbone). (UV-Vis (CH_2Cl_2), λ_{max}/nm (logε): 297 (4.54), 317 (4.30), 356 (3.30)

S1.2.5 [Ag(mqzt)(dppm)]$_2$ (5)

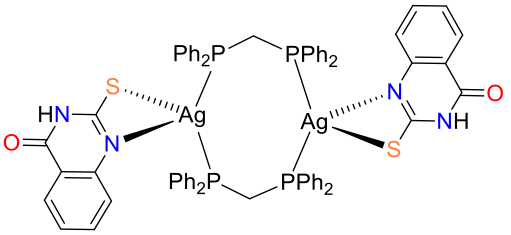

To an amount of 0.051 g (0.3 mmol) of $AgNO_3$ dissolved in 10 mL of CH_2Cl_2, dppm (0.115 g, 0.3 mmol) was added and contents were stirred at room temperature for 2 h in the dark. Then, a solution of $K^+(mqzt)^-$, obtained by the addition of 1.3 mL of 0.23 M methanolic solution of KOH into a solution of mqztH (0.054 g, 0.3 mmol) in 10 mL of CH_2Cl_2 was added dropwise and the reaction mixture was further stirred for 2 h in dark. After stirring, the resulting solution was let to cool at room temperature and then filtered off. The filtrate was layered with n-C_6H_{14} and colorless crystals of **5** were grown over a period of 7 days, which were collected. Crystals of **5** were dried under vacuum to afford the solvent-free product. Yield 0.037 g (19%). Anal. Calcd for $C_{66}H_{54}Ag_2N_4O_2P_4S_2$]: % C, 59.21; H, 4.07; N, 4.18. Found: % C, 59.61; H, 4.47; N, 4.58. FTIR (KBr, cm^{-1}): 516 (s), 548 (m), 589 (m), 695 (s), 749 (s), 765 (s), 910 (m), 965 (m), 998 (m), 1026 (m), 1072 (m), 1096 (s), 1229 (m), 1358 (s), 1436 (s), 1505 (m), 1598 (m), 1820 (w), 1892 (w), 1960 (w), 3055 (m). ^{1}H NMR (500 MHz, DMSO-d_6): δ (ppm) 12.09 (s, broad, 2H, mqzt, NH), 8.17-8.08 (m, 4H, mqzt), 7.85-7.84 (m, 16H, o-H, phenyl), 7.49-7.28 (m, 8H, p-H, phenyl), 7.16-7.00 (m, 16H, m-H, phenyl), 6.53 (s, 4H, mqzt), 3.61 (s, 4H, dppm, backbone). UV-Vis (CH_2Cl_2), λ/nm (logε): 296 (4.56), 319 (4.20), 340 (3.78).

S1.2.6 [Ag(mqzt)(xantphos)]$_2$ (6)

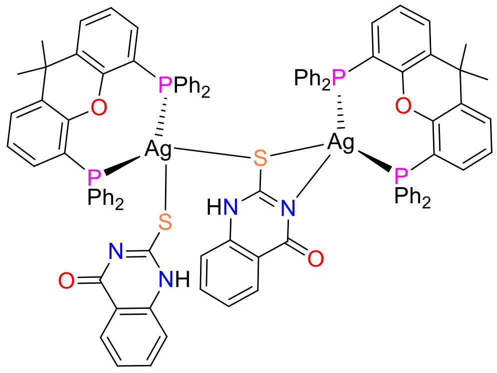

A portion of $AgNO_3$ (0.051 g, 0.3 mmol) was dissolved in 10 mL of CH_3CN, then xantphos (0.174 g, 0.3 mmol) was added, and the resulting solution was stirred at room temperature for 40 min in the dark. A solution of $K^+(mqzt)^-$, obtained by the addition of 1.5 mL of 0.2 M methanolic solution of KOH into a solution of mqztH (0.054 g, 0.3 mmol) in 10 mL of CH_2Cl_2, was added and the resulting mixture was further stirred in ambient conditions for 2 h. The resulting suspension was allowed to cool at room temperature and then filtered to remove a small amount of white solid. Large white crystals of **6** were formed upon layering of the filtrate with hexane, over a period of 20 days, which were collected. Crystals of **6** were dried under vacuum to afford the solvent-free product. Yield (Based on Ag): 0.020 g (8 %). Anal. Calcd for [$C_{94}H_{74}Ag_2N_4O_4P_4S_2$]: % C, 65.36; H, 4.32; N, 3.24. Found: % C, 64.21; H, 4.54; N, 3.24. FTIR (KBr, cm^{-1}): 510 (s), 553 (s), 588 (m), 692 (s), 720 (w), 742 (m), 762 (s), 890 (m), 963 (s), 999 (w), 1025 (m), 1057 (m),

1099 (w), 1185 (bs), 1239 (w), 1353 (w), 1435 (s), 1481 (m), 1501 (m), 1602 (m), 3053 (m). ^{1}H NMR (500 MHz, DMSO-d_6): δ (ppm) 12.11-11.75 (d, 2H, mqzt, N^2H), 7.80-7.78 (m, 2H, mqzt), 7.76-7.68 (m, 6H, mqzt), 7.35-7.32 (m, 16H, m-H, phenyl), 7.30-7.28 (m, 8H, p-H, phenyl), 7.26-7.19 (m, 16H, o-H, phenyl), 7.04 (s, 2H, xantphos backbone), 6.94-6.91 (t, 1H, xantphos backbone), 6.79-6.77 (d, 1H, xantphos backbone), 6.55-6.53 (m, 4H, xantphos backbone), 1.62 (s, 12H, CH_3). UV-Vis (CH_2Cl_2), λ/nm (logε): 295 (4.54), 356 (3.18).

S1.3 Synthesis and characterization of [1–25]@BaAlg, [1–50]@BaAlg and [1–25]@BaAlg hydrogels

S1.3.1 Synthesis of hydrogels

Solutions of complex **1** in DMSO at three concentrations (25, 50, and 100 μg mL^{-1}) were prepared and added to a 2% v/v sodium alginate solution. Each mixture was then added in small portions into a 1% v/v $BaCl_2$ solution resulting in the formation of hydrogel beads **[1]@BaAlg** (designated as **[1–25]@BaAlg**, **[1–50]@BaAlg**, and **[1–100]@BaAlg**, reflecting the initial concentration of complex **1** used in each case) via crosslinking of the alginate fibres by the Ba^{2+} ions and concomitant encapsulation of complex **1.**

S1.3.2 Loading capacity and encapsulation efficiency of hydrogels

Loading capacity (LC) of complex **1** into the **BaAlg** hydrogels (how much complex was loaded per unit mass of hydrogel) as well as their encapsulation efficiency (EE) (how much of the complex was successfully encapsulated) were calculated by suspending 0.5 g of the synthesized hydrogels into 10 mL of PBS solution. UV measurements at 295 nm were conducted, which correspond to the absorption band maximum of complex **1**. The calibration curve was created by diluting a stock solution of complex **1** (50 μg/mL) to the concentrations of 0.25, 0.5, 1, 3, 6, 12.5, 25, 50 μg/mL.

Loading capacity (LC) and encapsulation efficiency (EE) of complex **1** in the synthesized hydrogels were calculated based on the following equations:

$$LC\ (\%) = (W_{encapsulated\ complex} / W_{hydrogel})\times 100 \quad \text{(eq. 1)}$$

$$EE\ (\%) = (W_{encapsulated\ complex} / W_{total\ complex})\times 100 \quad \text{(eq. 2)}$$

$W_{total\ complex}$ = total mass of complex initially added

$W_{free\ complex}$ = mass of complex found in the supernatant

$W_{encapsulated\ complex} = W_{total\ complex} - W_{free\ complex}$

$W_{hydrogel}$ = mass of hydrogel

S1.4 Instrumentation

Elemental analyses were obtained on a PerkinElmer 240B elemental microanalyzer.

Fourier Transform Infrared (FTIR) spectra were recorded on a Thermo Scientific Nicolet iS20 spectrophotometer equipped with an Attenuated Total Reflection (ATR) accessory, in the region of 400–4000 cm^{-1}.

^{1}H NMR spectra were recorded in $CDCl_3$ solutions on an Agilent 500 spectrometer. Chemical shifts were reported as δ values using the solvent as internal standard.

UV-Vis electronic absorption spectra were recorded on a JASCO V-750 spectrophotometer.

Emission/excitation fluorescence spectra were recorded on a Hitachi F-7000 fluorescence spectrometer.

Cyclic voltammetry measurements were recorded on an Autolab electrochemical analyzer, using a carbon working electrode, a platinum counter electrode, and an Ag/AgCl electrode saturated with a KCl reference electrode in 8 mL of CH_3CN solutions with 0.1 M Bu_4NBF_4 as supporting electrolyte, with a scan rate of 0.1 V s^{-1}. Argon was used to purge all samples.

Thermogravimetric analysis (TGA) measurements were performed using a Netzsch STA 449F5 instrument (Netzsch Group, Selb, Germany). Pre-dried samples (5 mg) were placed in alumina crucibles and were heated under a 50 mL/min flow of N_2 and a heating rate of 10 K/min in the temperature range of 25-950 °C.

Dynamic light scattering (DLS) measurements for particle size determination was performed using a Litesizer 500 particle analyzer (Anton Parr, Austria).

Optical microscopy images of L929 cells were taken using a Nikon Eclipse TS-100 inverted optical microscope equipped with a Nikon DS-Fi3 microscope camera.

S1.5 Single-crystal X-ray diffraction analysis and calculation of steric effect of ligands

Single crystals of complexes **1-6** suitable for crystal structure analysis were mounted at room temperature on a Bruker Kappa APEX2 diffractometer equipped with a triumph monochromator using Mo Kα (λ = 0.71073 Å, source operating at 50 kV and 30 mA) radiation. Unit cell dimensions were determined and refined by using the angular settings of at least 176 high intensity reflections (>10σ(I)) in the range 11 < 2θ < 36°. Intensity data were recorded using φ and ω-scans. All crystals presented no decay during the data collection. The frames collected for each crystal were integrated with the Bruker SAINT Software package [59], using a narrow–frame algorithm. Data were corrected for absorption using the numerical method (SADABS) based on crystal dimensions [59]. The structure was solved using the SUPERFLIP package [60], incorporated in Crystals. Data refinement (full–matrix least–squares methods on F2) and all subsequent calculations were carried out using the Crystals version 14.61 build 6236 program package [61]. All non–hydrogen non–disordered atoms in all compounds were refined anisotropically. For the disordered atoms in complexes **1**, **2** and **6**, their occupation factors were first detected under fixed isotropic thermal parameters. Afterwards all were refined with fixed occupation factors, anisotropically in the case of the sulfur atom in compound 1 and isotropically in the case of disordered acetonitrile/dichloromethane solvents of complexes **1**, **2** and **6**. Hydrogen atoms riding on non-disordered parent atoms were located from difference Fourier maps and refined at idealized positions riding on the parent atoms with isotropic displacement parameters Uiso(H) = 1.2Ueq(C) or 1.5Ueq(-NH) and at distances C–H 0.95 Å and N–H 0.83 Å. All methyl and amine hydrogen atoms were allowed to rotate. Hydrogen atoms riding on disordered methyl/methylene carbon atoms were positioned geometrically to their parent atoms. All molecular plots and packing diagrams were prepared using Mercury software [61].

To quantify the steric effects of phosphines PPh_3, xantphos, DPEPhos in complexes **1-6**, their respective percent buried volume parameters ($\%V_{bur}$) were determined. The calculations were performed using the X-ray crystallographic data of complexes **1-6** and the SambVca 2.1 web application.[62] The following conditions were used for each calculation: sphere radius = 3.5 Å, d(Ag−P) = 2.28 Å, H atoms not included, and Bondi radii scaled by 1.17. The calculated $\%V_{bur}$ parameters are listed in Table S8.

S1.6 In vitro antibacterial activity studies

S1.6.1 Broth microdilution assay

The antibacterial activity of the complexes **1-6** against two bacterial strains, *E. coli* and *S. aureus*, was monitored following the method described by J. M. Andrews [63], using progressive double dilutions in MMS contained the concentrations of 100, 50, 25 and 12.5 μg mL^{-1} of the complexes in DMSO. The growth of bacteria was monitored by measuring the turbidity of the culture in the tubes [64]. The cultivation media used for antibacterial activity tests were: (i) the Luria–Bertani broth containing 1% w/v tryptone, 0.5% w/v NaCl and 0.5% w/v yeast extract and (ii) the minimal medium salts broth containing 0.5% w/v glucose, 8.5 g/L $Na_2HPO_4{\cdot}2H_2O$, 3 g/L K_2HPO_4, 1 g/L NH_4Cl, 0.5 g/L NaCl, 1 mM $MgSO_4{\cdot}7H_2O$, 0.1 mM $CaCl_2$, 800 ug/mL Leucine, 10 ug/mL Thiamine. The pH of the media was adjusted to 7.0.

S1.7 Mechanistic investigations

S1.7.1 In vitro Glutathione reductase (GR) and Thioredoxin reductase (TrxR) inhibition assay

The TrxR (*E. coli*) inhibition assay was performed according to previously published procedures. The assay is partly based on the procedure developed by Lu et al. described in and makes use of the reduction in DTNB (5,5′-dithiobis-(2-nitrobenzoic acid) [65,66]. Stock solutions of *E. coli* TrxR (purchased from Abcam and diluted to the specific activity of 0.2 U/mL) and *E. coli* thioredoxin (Trx, purchased from Merck and diluted to 156 μg/mL) or *E. coli* GR (purchased from Antibodies online and diluted to the specific activity of 4.2 U/ml,) and oxidized glutathione (GSSG, purchased from Merck and diluted to 0.28 mM) were prepared in distilled water. Stock solutions of the test compounds (2 mM) were prepared in DMSO and serially diluted with TE buffer (Tris-HCl 50 mM, EDTA 1 mM, pH 7.5). 20 μL of these solutions or TE buffer without the test compounds (positive control) were mixed with the TrxR solution (10 μL), the Trx solution (10 μL) and 100 μl of NADPH (200 μM) in TE buffer in a well on a 96-well plate. As a blank control, 200 μM NADPH in TE buffer (100 μL) mixed with a DMSO / buffer mixture (40 μL) was used (final concentrations of DMSO: 0.5% v/v). The plate was incubated for 75 min at 25 °C with moderate shaking. After incubation, 100 μL of a reaction mixture (TE buffer containing 200 μM NADPH and 5 mM DTNB) was added to each well to initiate the reaction. After thorough mixing, the formation of 5-TNB was monitored by a microplate reader at 405 nm in 35 s intervals (10 measurements). The values were corrected by subtraction of the blank solution absorption values. The increase in concentration of 5-TNB followed a linear trend ($r^2 \geq 0.990$) and the enzymatic activities were calculated as the gradients (increase in absorbance per second) thereof. Absence of interference with the assay components was confirmed by a negative control experiment for each test compound, where the highest test compound concentration was used and the enzyme solution was replaced by TE buffer. The inhibition is presented as the mean IC_{50} values and standard deviations obtained in three independent experiments.

S2 RESULTS

S2.1 Single crystal X-ray diffraction analysis

Table S1. Crystal data, data collection, and refinement parameters for complexes **1-6**.

	**1**·CH_3CN	**2**· CH_3CN	**3**·$2CH_2Cl_2$
Chemical formula	$C_{46}H_{39}AgClN_3OP_2S$	$C_{49}H_{41}AgClN_3O_2P_2S$	$C_{54}H_{44}Ag_2Cl_4N_4O_2P_2S_2$
Formula weight	887.17	941.22	1264.59
Crystal system	Triclinic	Monoclinic	Monoclinic
Space group	*P*-1	$P2_1/c$	$P2_1/n$
Temperature (K)	295	295	295
Unit cell parameters			
a (Å)	11.3011 (6)	18.7253 (12)	16.4193 (16)
b (Å)	12.9262 (7)	10.0103 (7)	10.2328 (9)
c (Å)	16.3177 (8)	25.4012 (16)	16.6164 (17)
α (°)	74.6819 (14)	90	90
β (°)	79.3742 (15)	107.128 (2)	110.826 (3)
γ (°)	70.7481 (15)	90	90
V (Å^3)	2158.0 (2)	4550.2 (5)	2609.4 (4)
Z	2	4	2
Radiation type, λ (Å)	Mo $K\alpha$	Mo $K\alpha$	Mo $K\alpha$
Absorption coefficient (mm^{-1})	0.69	0.66	1.14
Crystal size (mm)	0.21 × 0.17 × 0.12	0.21 × 0.17 × 0.12	0.19 × 0.16 × 0.16
Diffractometer	Bruker Kappa Apex2	Bruker Kappa Apex2	Bruker Kappa Apex2
Absorption correction	Numerical Analytical Absorption (De Meulenaer & Tompa, 1965)	Numerical Analytical Absorption (De Meulenaer & Tompa, 1965)	Numerical Analytical Absorption (De Meulenaer & Tompa, 1965)
T_{min}, T_{max}	0.89, 0.92	0.89, 0.92	0.83, 0.83
Number of measured, independent and observed [I> 2.0σ(I)] reflections	33850, 8320, 6592	43987, 8583, 6434	23161, 5023, 3544
R_{int}	0.026	0.041	0.039
(sin θ/λ)$_{max}$ (Å^{-1})	0.616	0.610	0.616
$R[F^2>2\sigma(F^2)]$, $wR(F^2)$, S	0.045, 0.078, 1.00	0.045, 0.068, 1.00	0.044, 0.083, 1.00
No. of reflections	6592	6434	3544
No. of parameters	502	537	316
No. of restraints	20	20	2
H-atom treatment	H-atom parameters constrained	H-atom parameters constrained	H-atom parameters constrained
$\Delta\rho_{max}$, $\Delta\rho_{min}$ (e Å^{-3})	1.25, −0.87	0.79, −0.54	0.66, −0.91

(Continued)

(Continued)

	**4**·C_6H_{14}	**5**	**6**·$2CH_2Cl_2$
Chemical formula	$C_{94}H_{80}Ag_2N_4O_4P_4S_2$	$C_{66}H_{54}Ag_2N_4O_2P_4S_2$	$C_{96}H_{78}Ag_2Cl_4N_4O_4P_4S_2$
Formula weight	1733.46	1338.94	1897.27
Crystal system	Triclinic	Triclinic	Monoclinic
Space group	*P*-1	*P*-1	$P2_1/n$
Temperature (K)	295	295	295
Unit cell parameters			
a (Å)	12.6398 (10)	11.4092 (4)	18.505 (13)
b (Å)	13.5662 (11)	11.5056 (4)	19.173 (12)
c (Å)	15.0232 (13)	12.4521 (6)	28.402 (19)
α (°)	63.168 (4)	100.106 (3)	90
β (°)	69.701 (5)	93.046 (2)	103.821 (16)
γ (°)	67.704 (4)	107.874 (2)	90
V (Å^3)	2078.2 (3)	1521.53 (11)	9785 (11)
Z	1	1	4
Radiation type, *λ* (Å)	Mo *K*α	Mo *K*α	Mo *K*α
Absorption coefficient (mm^{-1})	0.65	0.86	0.67
Crystal size (mm)	0.12 × 0.06 × 0.03	0.24 × 0.18 × 0.15	0.18 × 0.17 × 0.11
Diffractometer	Bruker Kappa Apex2	Bruker Kappa Apex2	Bruker Kappa Apex2
Absorption correction	Numerical Analytical Absorption (De Meulenaer & Tompa, 1965)	Numerical Analytical Absorption (De Meulenaer & Tompa, 1965)	Numerical Analytical Absorption (De Meulenaer & Tompa, 1965)
T_{min}, T_{max}	0.96, 0.98	0.86, 0.88	0.89, 0.93
Number of measured, independent and observed [I> 2.0σ(I)] reflections	35328, 7839, 5472	26132, 5856, 4275	80414, 18522, 10902
R_{int}	0.058	0.053	0.043
(sin θ/λ)$_{max}$ (Å^{-1})	0.610	0.615	0.615
$R[F^2>2σ(F^2)]$, $wR(F^2)$, S	0.056, 0.094, 1.00	0.032, 0.057, 1.00	0.060, 0.093, 1.00
No. of reflections	5472	4275	10902
No. of parameters	496	361	1063
No. of restraints	6		15
H-atom treatment	H-atom parameters constrained	H-atom parameters constrained	H-atom parameters constrained
$Δρ_{max}$, $Δρ_{min}$ (e Å^{-3})	0.92, −0.55	0.34, −0.30	0.89, −0.94

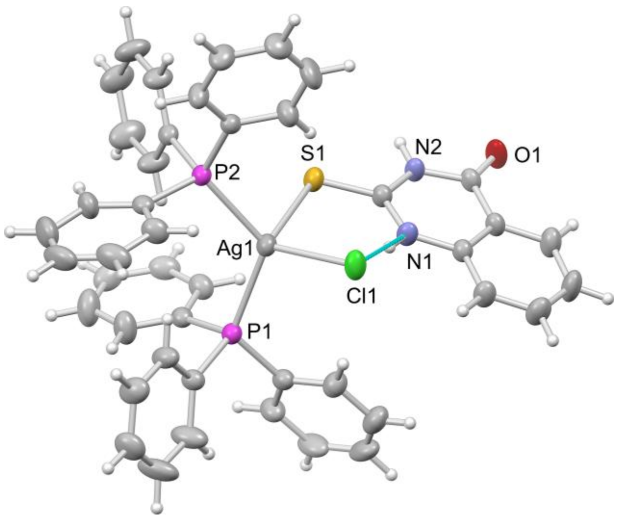

Figure S1. Crystal structure of [AgCl(mqztH)(PPh_3)$_2$] (**1**). Atoms are presented as thermal ellipsoids at the 35% probability level. Hydrogen atoms are shown as spheres of arbitrary radius. Intramolecular hydrogen bonding interaction NH···Cl is also shown (blue color dotted line).

Table S2. Selected bond lengths (Å) and angles (°) for complex **1**.

Bond lengths (Å)			
Ag1–P1	2.483 (1)	Ag1–S1	2.786 (2)
Ag1–P2	2.466 (1)	Ag1–Cl1	2.556 (1)
N1···Cl1	3.082		
**Bond angles (°)**			
P1–Ag1–P2	121.58 (3)	P1–Ag1–Cl1	109.01 (4)
P1–Ag1–S1	108.3 (6)	P2–Ag1–Cl1	116.39 (4)
P2–Ag1–S1	98.4 (4)	S1–Ag1–Cl1	98.63 (4)

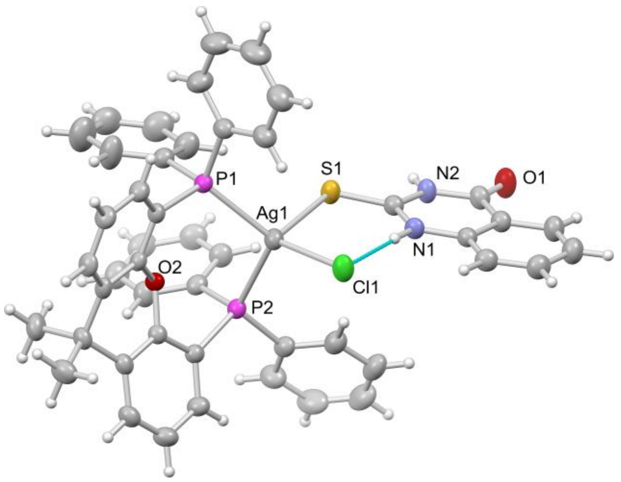

Figure S2. Crystal structure of [AgCl(mqztH)(xantphos)] (**2**). Atoms are presented as thermal ellipsoids at the 35% probability level. Hydrogen atoms are shown as spheres of arbitrary radius. Intramolecular hydrogen bonding interaction NH···Cl is also shown (blue color dotted line).

Table S3. Selected bond lengths (Å) and angles (°) for complex **2**.

Bond lengths (Å)			
Ag1–P1	2.470 (1)	Ag1–S1	2.663 (1)
Ag1–P2	2.505 (1)	Ag1–Cl1	2.543(1)
N1···Cl1	3.109		
**Bond angles (°)**			
P1–Ag1–P2	109.42 (3)	P1–Ag1–Cl1	119.84 (3)
P1–Ag1–S1	110.05 (4)	P2–Ag1–Cl1	114.27 (3)
P2–Ag1–S1	99.52 (3)	S1–Ag1–Cl1	101.36 (3)

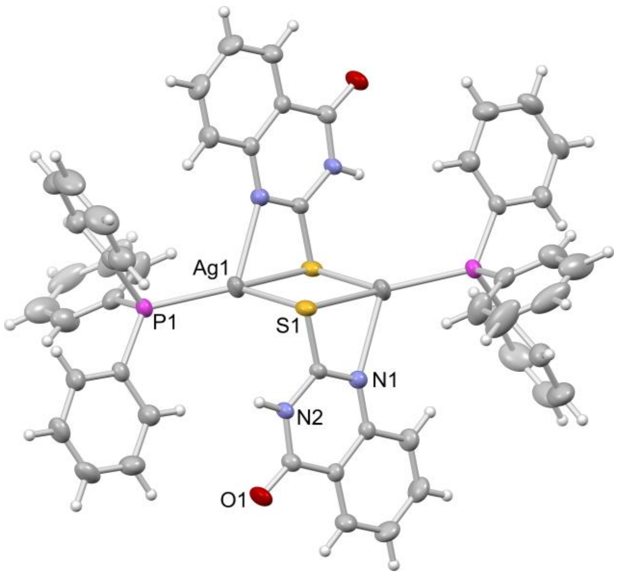

Figure S3. Crystal structure of $[Ag(mqzt)(PPh_3)]_2$ (**3**). Atoms are presented as thermal ellipsoids at the 35% probability level. Hydrogen atoms are shown as spheres of arbitrary radius.

Table S4. Selected bond lengths (Å) and angles (°) for complex **3**.

Bond lengths (Å)			
Ag1–P1	2.389 (1)	Ag1–S1^{i}	2.776 (1)
Ag1–S1	2.557 (1)	Ag1–N1	2.430 (4)
Ag1···Ag1^{i}	3.701		
**Bond angles (°)**			
P1–Ag1–S1	131.09 (4)	S1^{i}–Ag1–S1	92.23 (4)
S1^{i}–Ag1–P1	133.03 (4)	N1^{i}–Ag1–S1	100.71 (9)
N1^{i}–Ag1–P1	116.21 (9)	S1^{i}–Ag1–N1^{i}	60.50(9)

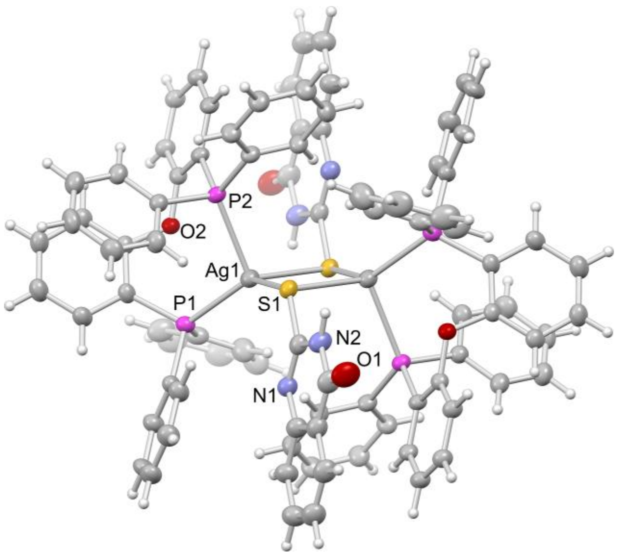

Figure S4. Crystal structure of [Ag(mqzt)(DPEPhos)]$_2$ (**4**). Atoms are presented as thermal ellipsoids at the 35% probability level. Hydrogen atoms are shown as spheres of arbitrary radius.

Table S5. Selected bond lengths (Å) and angles (°) for complex **4**.

Bond lengths (Å)			
Ag1–P1	2.473 (1)	Ag1–S1^i	2.638 (2)
Ag1–P2	2.561 (2)	Ag1···Ag1^i	3.343 (1)
Ag1–S1	2.620 (2)		
**Bond angles (°)**			
P1–Ag1–P2	109.53 (5)	S1^i–Ag1–P1	111.43 (5)
P1–Ag1–S1	132.06 (5)	S1^i–Ag1–P2	110.06 (5)
P2–Ag1–S1	90.10 (4)	S1^i–Ag1–P1	111.43 (5)

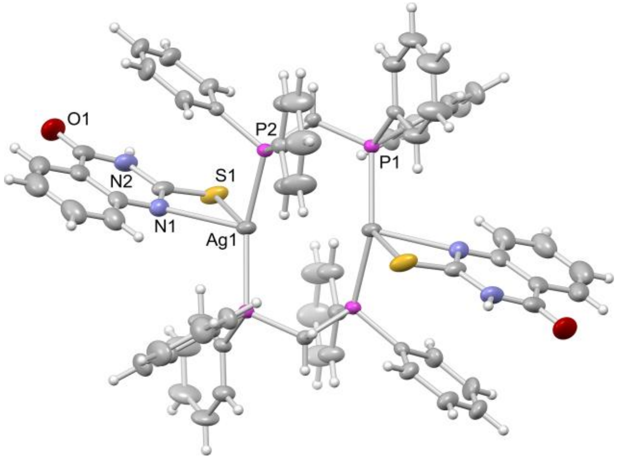

Figure S5. Crystal structure of [Ag(mqzt)(dppm)]$_2$ (**5**). Atoms are presented as thermal ellipsoids at the 35% probability level. Hydrogen atoms are shown as spheres of arbitrary radius.

Table S6. Selected bond lengths (Å) and angles (°) for complex **5**.

Bond lengths (Å)			
Ag1–P1	2.410 (1)	Ag1–S1	2.665 (1)
Ag1–P2	2.452 (1)	Ag1–N1	2.520 (3)
Ag1···Ag1^i	3.557		
**Bond angles (°)**			
P2^i–Ag1–P1	149.29 (3)	P2^i–Ag1–N1	91.81 (7)
P2^i–Ag1–S1	94.07 (3)	P1–Ag1–N1	103.92 (7)
P1–Ag1–S1	116.60 (3)	S1–Ag1–N1	60.97 (8)

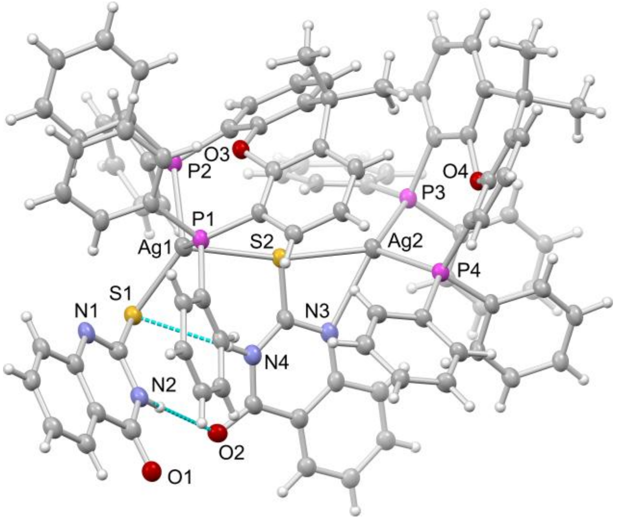

Figure S6. Crystal structure of [Ag(mqzt)(xantphos)]$_2$ (**6**). Atoms are presented as thermal ellipsoids at the 35% probability level. Hydrogen atoms are shown as spheres of arbitrary radius. Intramolecular hydrogen bonding interactions NH···S and NH···O are also shown (blue color dotted lines).

Table S7. Selected bond lengths (Å) and angles (°) for complex **6**.

Bond lengths (Å)			
Ag1–P1	2.498 (2)	Ag1–S1	2.422 (2)
Ag1–P2	2.442 (2)	Ag1–S2	2.786 (2)
Ag2–P3	2.473 (2)	Ag2–S2	2.568 (2)
Ag2–P4	2.492 (2)	Ag2–N3	2.549 (5)
N4(H)···S1	3.349	N2(H)···O2	3.039
**Bond angles (°)**			
P1–Ag1–P2	108.40 (7)	P2–Ag1–S2	93.48 (8)
P1–Ag1–S1	120.92 (7)	S1–Ag1–S2	107.02 (7)
P2–Ag1–S1	119.96 (7)	P3–Ag2–P4	110.62 (7)
P1–Ag1–S2	101.47 (6)	P3–Ag2–S2	129.66 (6)
P4–Ag2–S2	117.46 (7)	P3–Ag2–N3	122.60 (12)

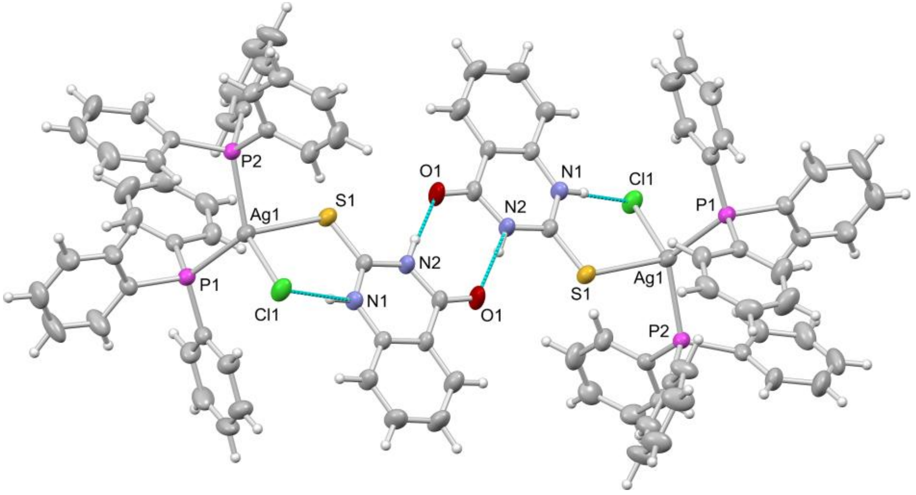

Figure S7. View of the intra- and intermolecular H-bonding interactions (blue color dotted lines) developed between neighboring [AgCl(mqztH)(PPh_3)$_2$] (**1**) molecules in the solid state (N1(H)···Cl1 = 3.082 Å and N2(H)···O1 = 2.831 Å). Atoms are presented as thermal ellipsoids at the 35% probability level. Hydrogen atoms are shown as spheres of arbitrary radius.

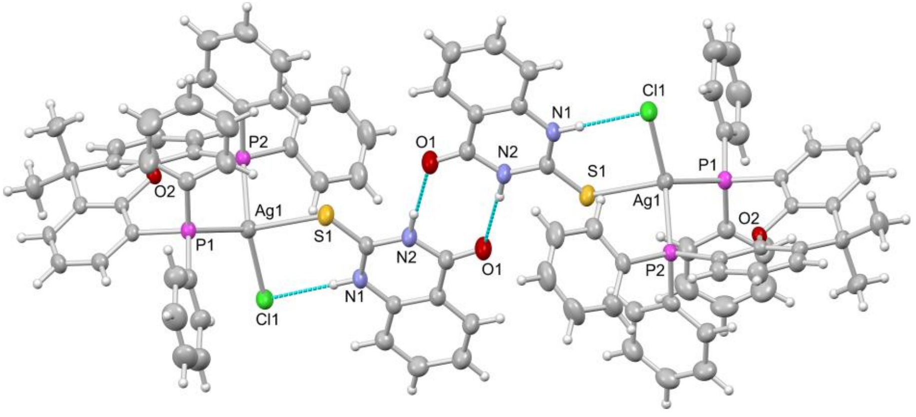

Figure S8. View of the intra- and intermolecular H-bonding interactions (blue color dotted lines) developed between neighboring [AgCl(mqztH)(xantphos)] (**2**) molecules in the solid state (N1(H)···Cl1 = 3.109 Å and N2(H)···O1 = 2.861 Å). Atoms are presented as thermal ellipsoids at the 35% probability level. Hydrogen atoms are shown as spheres of arbitrary radius.

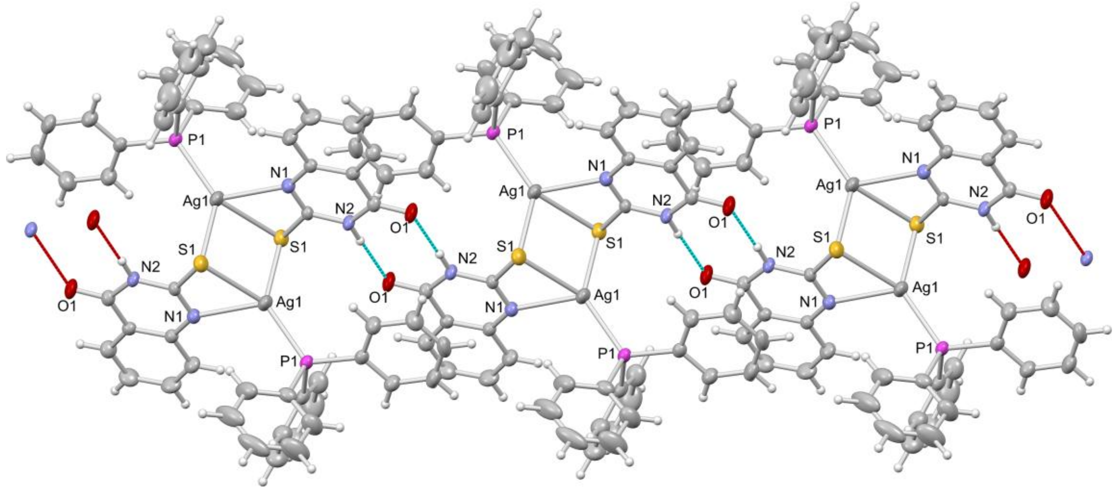

(a)

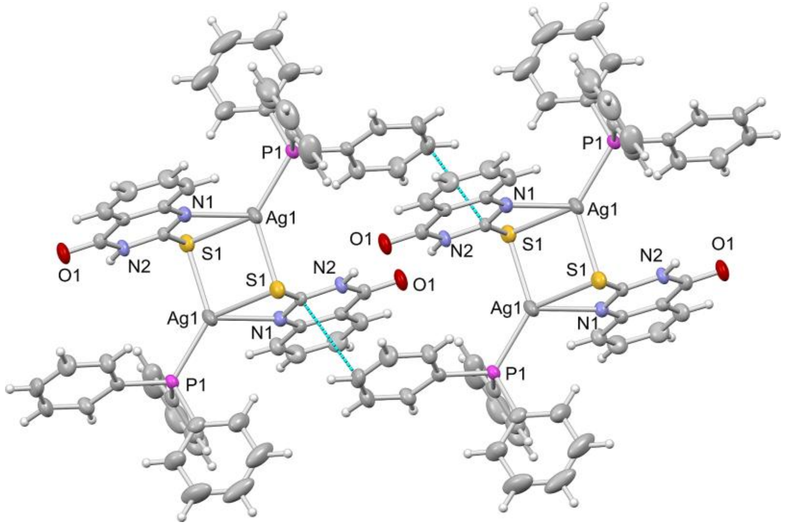

(b)

Figure S9. (a) View of the intermolecular H-bonding interactions (blue color dotted lines) developed between neighboring $[Ag(mqzt)(PPh_3)]_2$ (**3**) molecules in the solid state (N2(H)···O1 = 2.897 Å). (b) View of the intermolecular π-π interactions (blue color dotted lines) developed between neighboring $[Ag(mqzt)(PPh_3)]_2$ (**3**) molecules in the solid state. Atoms are presented as thermal ellipsoids at the 35% probability level. Hydrogen atoms are shown as spheres of arbitrary radius.

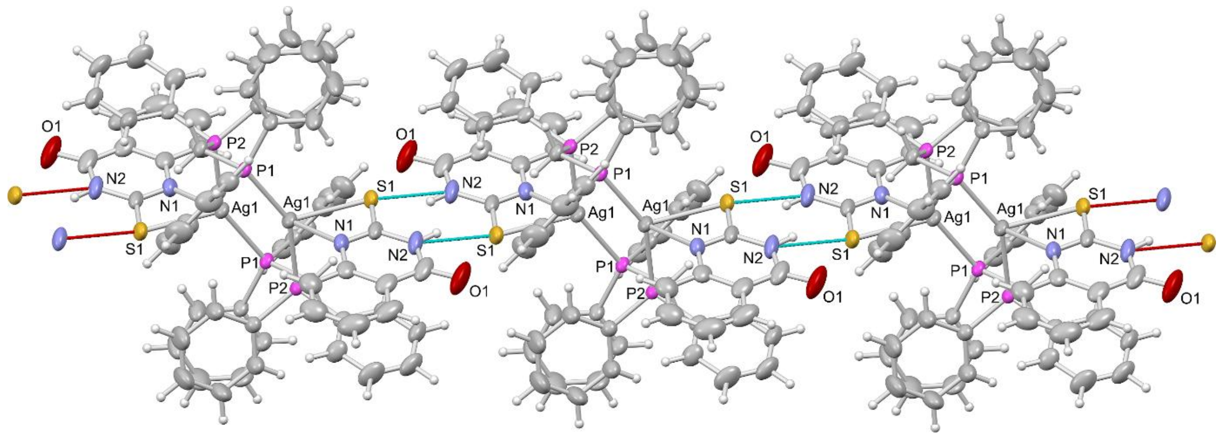

Figure S10. View of the intermolecular H-bonding interactions (blue color dotted lines) developed between neighboring $[Ag(mqzt)(dppm)]_2$ (**5**) molecules in the solid state (N2(H)···S1 = 3.260 Å). Atoms are presented as thermal ellipsoids at the 35% probability level. Hydrogen atoms are shown as spheres of arbitrary radius.

Table S8. %V_{bur} for phosphines PPh_3, xantphos, DPEPhos in complexes **1-6**

**Complex**	**Phosphine**	**%V_{bur}**
[AgCl(mqztH)(PPh_3)$_2$] (**1**)	PPh_3	39.8
[AgCl(mqztH)(xantphos)] (**2**)	xantphos	60.9
[Ag(mqzt)(PPh_3)]$_2$ (**3**)	PPh_3	33.3
[Ag(mqzt)(DPEPhos)]$_2$ (**5**)	DPEPhos	58.7
[Ag(mqzt)(xantphos)]$_2$ (**6**)	xantphos	56.6

%V_{bur} values were calculated using SambVca 2.1 web application with spherical radius = 3.5 Å, bondi radii scaled by 1.17, distance of the coordination point from the centre of the sphere = 0.0, mesh spacing for numerical integration = 0.10 and P−Ag bond length = 2.28 Å.

S2.2 FTIR spectroscopy

Table S9. Characteristic vibration bands in the FTIR spectra of complexes **1-6**.

	**ν(N–H) / cm^{-1}**	**ν (thioamide group) / cm^{-1}**			
		**I**	**II**	**III**	**IV**
[AgCl(mqztH)(PPh_3)$_2$] (**1**)	3052	1554	1266	1161	695
[AgCl(mqztH)(xantphos)] (**2**)	3054	1552	1264	1165	694
[Ag(mqzt)(PPh_3)]$_2$ (**3**)	3052	1568	1262	1164	693
[Ag(mqzt)(dppm)]$_2$ (**4**)	3050	1563	1250	1155	694
[Ag(mqzt)(DPEPhos)]$_2$ (**5**)	3055	1559	1230	1162	694
[Ag(mqzt)(xantphos)]$_2$ (**6**)	3051	1521	1229	1152	692

S2.3 ^{1}H NMR spectroscopy

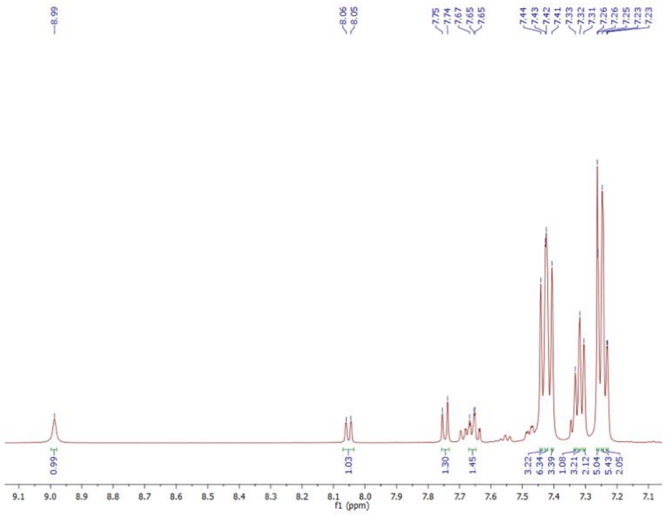

Figure S11. ^{1}H NMR spectrum of complex **1** in DMSO-d_6.

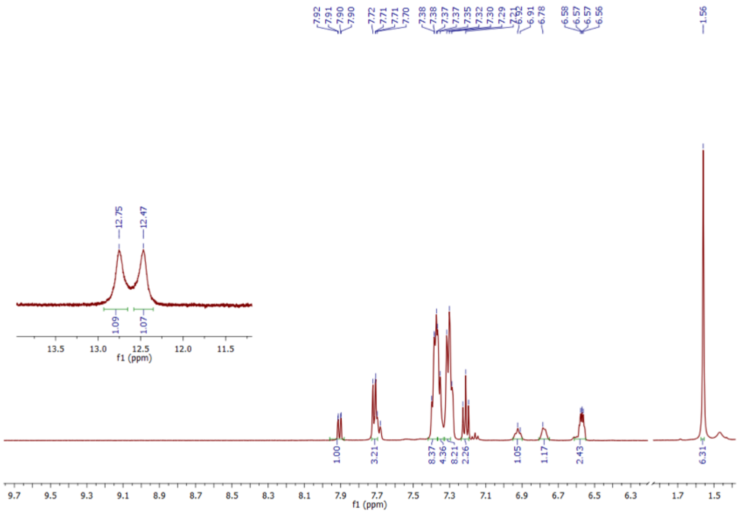

Figure S12. ^{1}H NMR spectrum of complex **2** in DMSO-d_6.

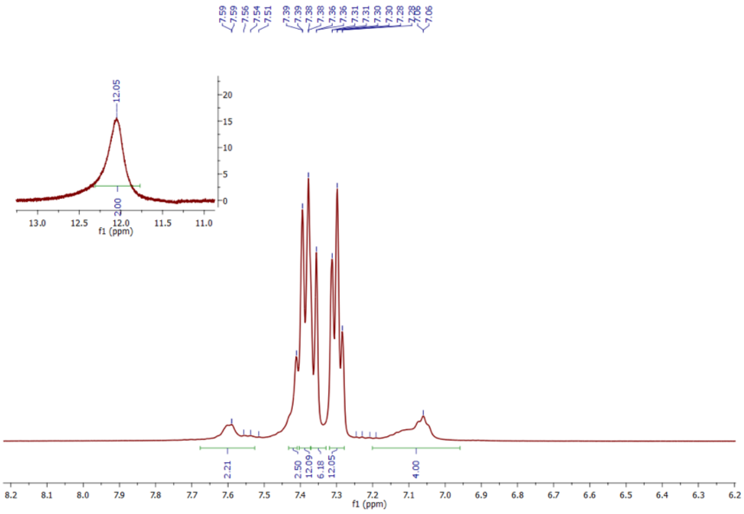

Figure S13. ^{1}H NMR spectrum of complex **3** in DMSO-d_6.

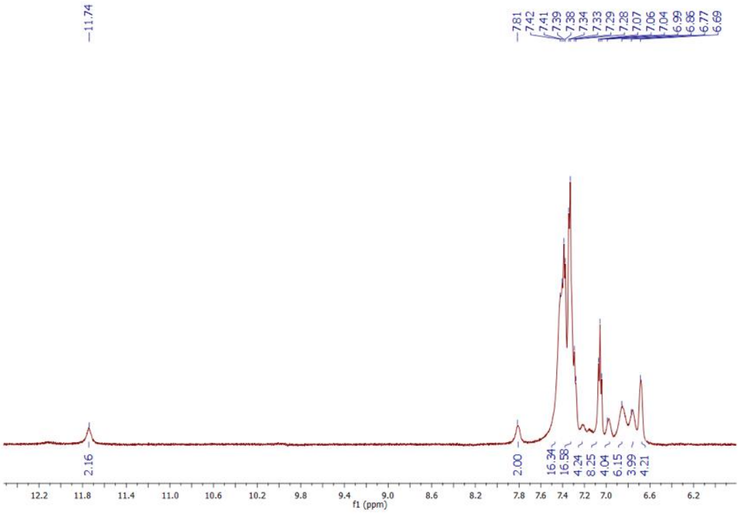

Figure S14. ^{1}H NMR spectrum of complex **4** in DMSO-d_6.

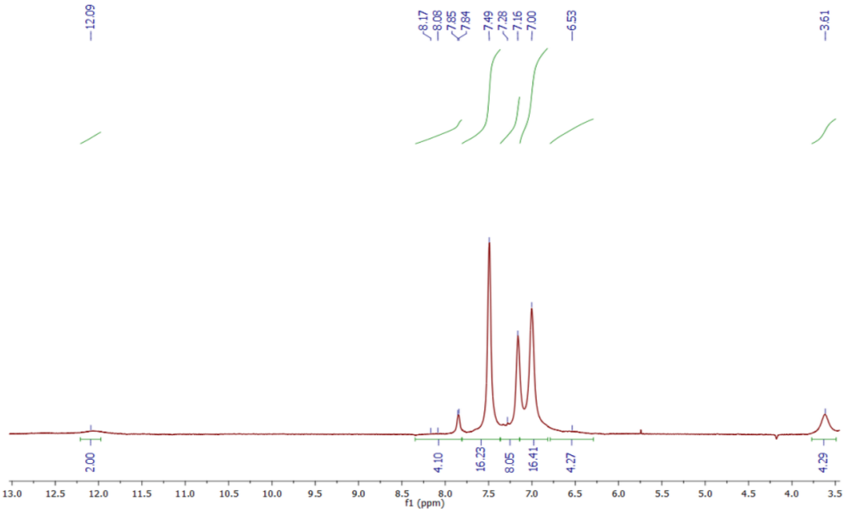

Figure S15. ^{1}H NMR spectrum of complex **5** in DMSO-d_6.

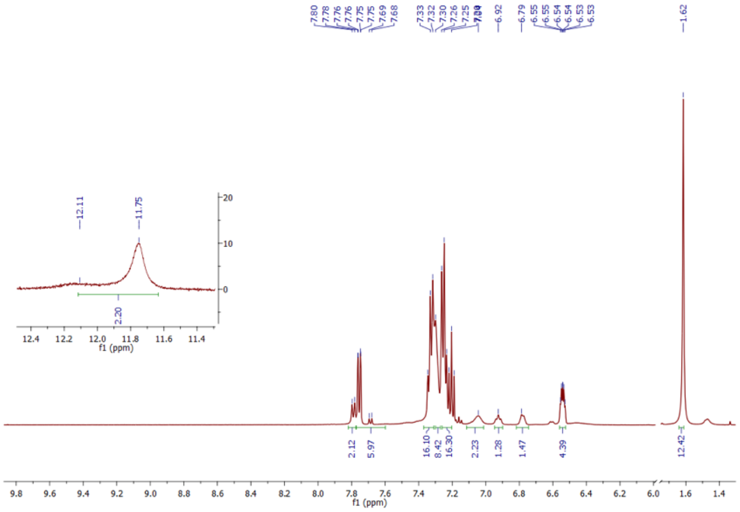

Figure S16. ^{1}H NMR spectrum of complex **6** in DMSO-d_6.

S2.4 Stability studies

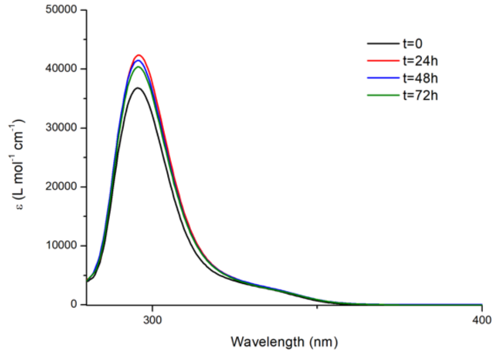

1

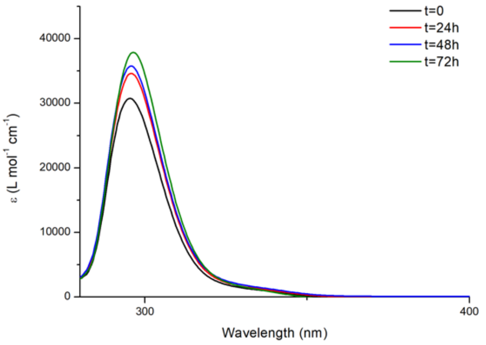

2

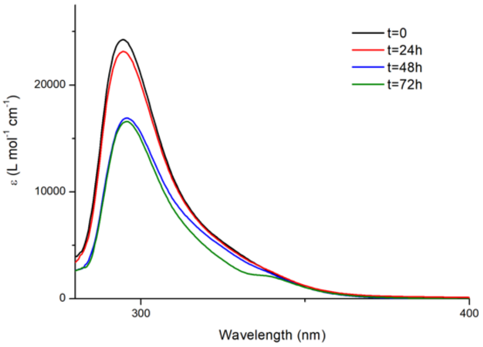

3

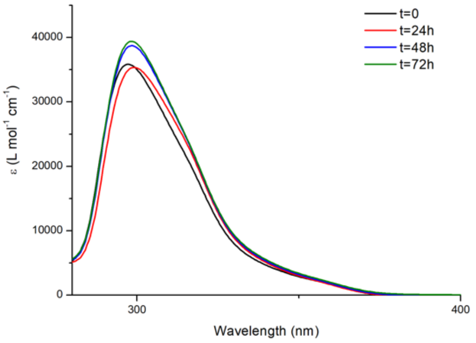

4

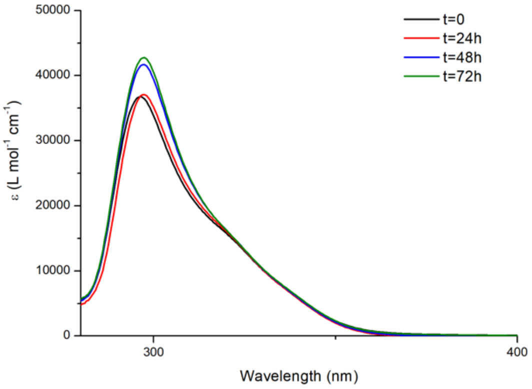

5

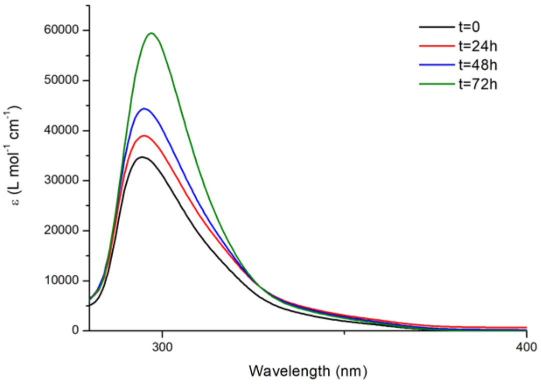

6

Figure S17. UV-vis absorption spectra of complexes **1-6** in DMSO (5×10^{-4} M) at 0, 24, 48 and 72h time intervals.

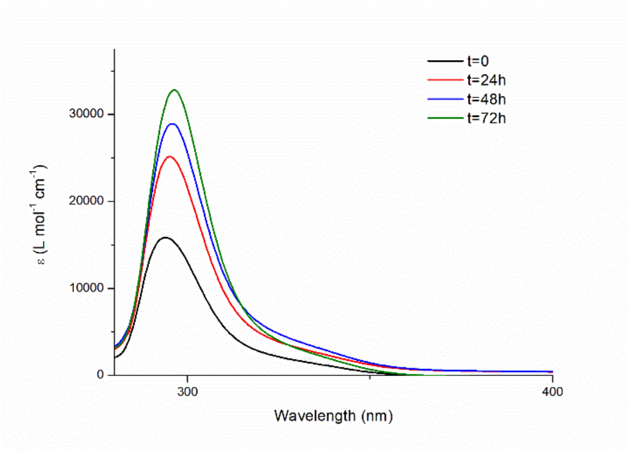

1

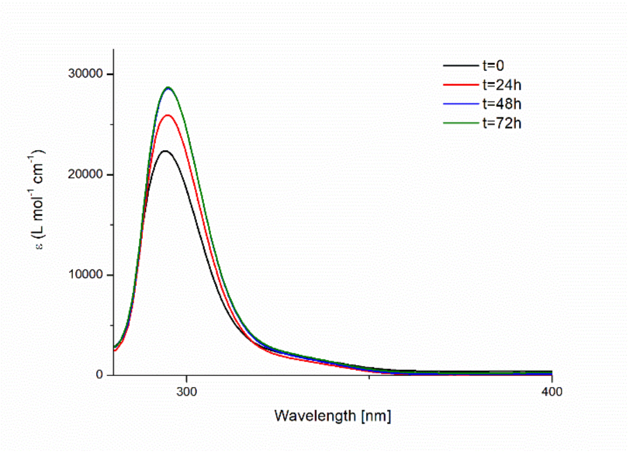

2

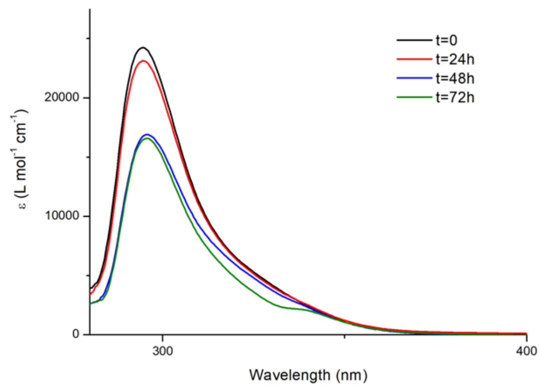

3

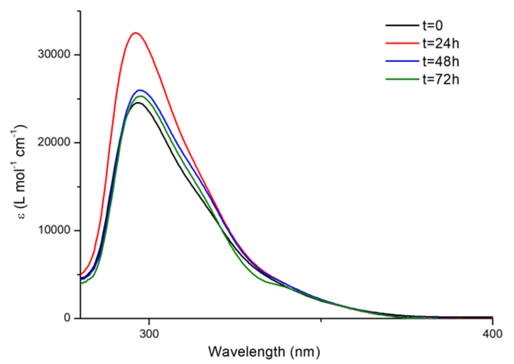

4

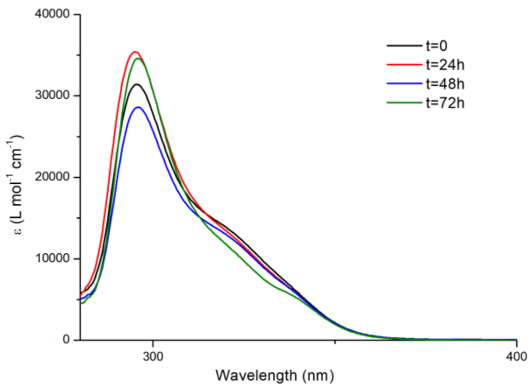

5

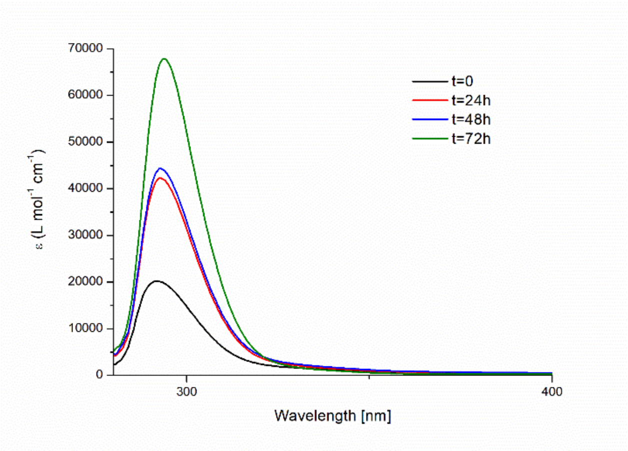

6

Figure S18. UV-vis absorption spectra of complexes **1-6** in DMSO/ddH_2O (5:1 v/v) mixtures (5×10^{-5} M) at 0, 24, 48 and 72h time intervals.

S2.5 Dynamic Light Scattering studies

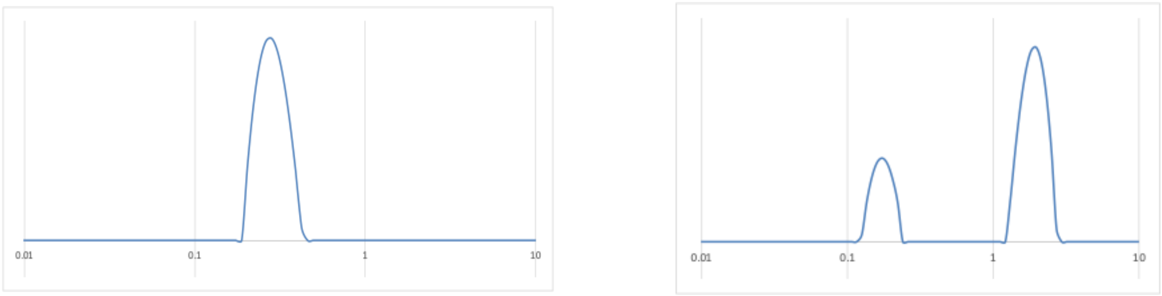

(a) (b)

Figure S19. Hydrodynamic average particle diameter distribution profiles of complex **1** in DMSO (5×10^{-4} M) solution determined by DLS measurements at 3 h (a) and 24 h (b) time intervals.

S2.6 In vitro antibacterial activity studies

Table S10. In vitro antibacterial activity of complexes **1**-**6**, and their ligands mqztH, PPh_3, DPEphos, xantphos and dppm in free form, against *E. coli* and *S. aureus* bacterial strains, expressed as half-minimum inhibitory concentration (IC_{50}) values (μg/mL) provided by a nonlinear curve fit-growth/sigmoidal-dose response on the experimental optical density data. Values are expressed as mean ± standard deviation (SD) of three replicate measurements (with the exception of values higher than 100 μg/mL). Ampicillin was used as reference compound with MIC values of 25 (71.6 μM) and 30 μg mL^{-1} (85.8 μM) for *E. coli* and *S. aureus* bacterial strains, respectively.

	E. coli	***S. aureus***	
	**IC_{50} μg mL^{-1} (μM)**	**MIC μg mL^{-1} (μM)**	**IC_{50} μg mL^{-1} (μM)**
[AgCl(mqztH)(PPh_3)$_2$] (**1**)	63.3 (±1.9) (75)	50 (59)	4.2 (±1.4) (4.9)
[AgCl(mqztH)(xantphos)] (**2**)	>100	>100	39.7 (±1.2) (6)
[Ag(mqzt)(dppm)]$_2$ (**4**)	>100	100 (74.6)	4.3 (±1.5) (3.2)
[Ag(mqzt)(DPEPhos)]$_2$ (**5**)	>100	100 (60.6)	6.7 (±1.9) (4.1)
[Ag(mqzt)(xantphos)]$_2$ (**6**)	>100	>100	15.7 (±4.3) (13)
mqztH	>100	>100	>100
PPh_3	>100	>100	>100
DPEPhos	>100	>100	>100
xantphos	>100	>100	>100
dppm	>100	>100	>100

S2.7 Thermogravimetric analysis

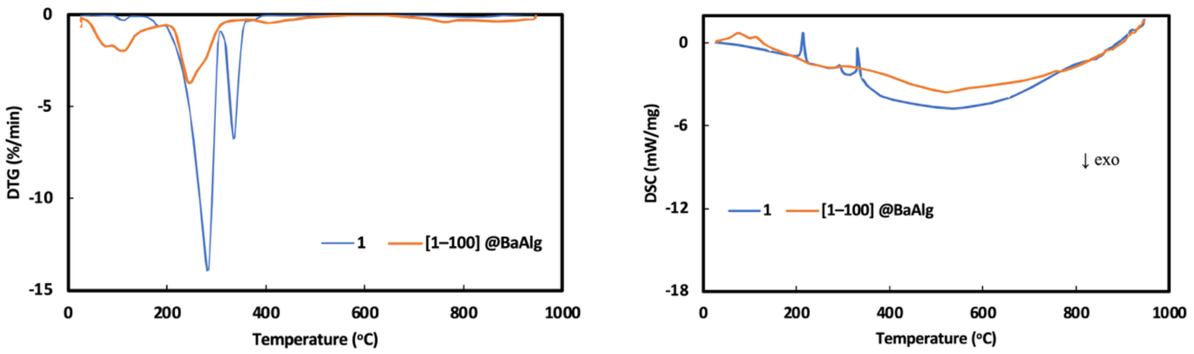

(a) (b)

Figure S20. Thermogravimetric analysis data of complex **1** in free form and **[1–100]@BaAlg** hydrogel.

Table S11. Thermogravimetric analysis data of complex **1** in free form and **[1–100]@BaAlg** hydrogel showing mass loss (%) at the corresponding temperature ranges for under N_2 atmosphere.

	T_{onset} (ºC)	$T_{DTG, max}$ (ºC)	T_{end} (ºC)	Mass loss temperature range (ºC)	Mass loss (%)	T_{onset} (ºC)	$T_{DTG, max}$ (ºC)	T_{end} (ºC)	Mass loss temperature range (ºC)	Mass loss (%)	Residual mass, 950ºC (%)
**1**	252	283	295	150-308	66.42	327	335	343	308-400	17.01	13.47
**[1–100]@BaAlg**	72	106		26-200	16.31	229	245	-	200-500	28.92	46.06

Measurements' conditions: 25-950ºC, 10 ºC/min, 50 cc/min, N_2, NETZSCH STA 449F5

S2.8 Electrochemical studies

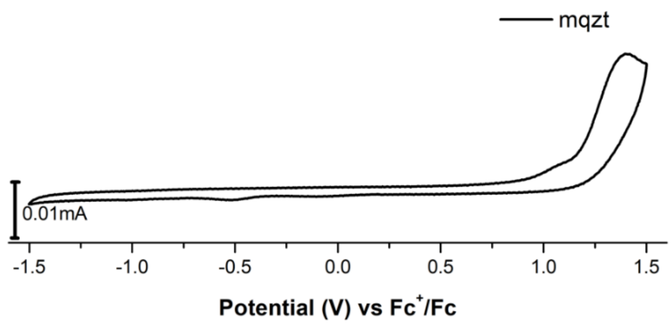

Figure S21. Cyclic voltammogram of mqztH in CH_3CN.

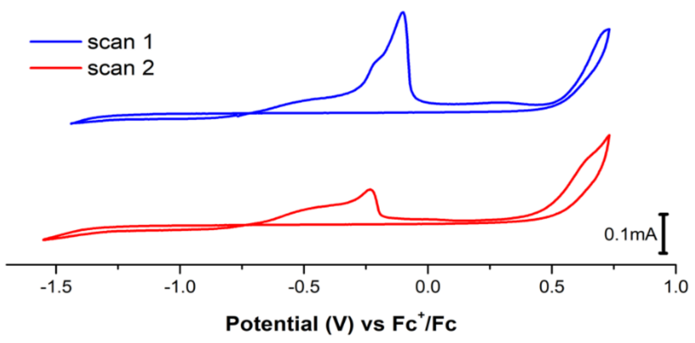

Figure S22. Consecutive cyclic voltammetry scans of complex **1** in CH_3CN.

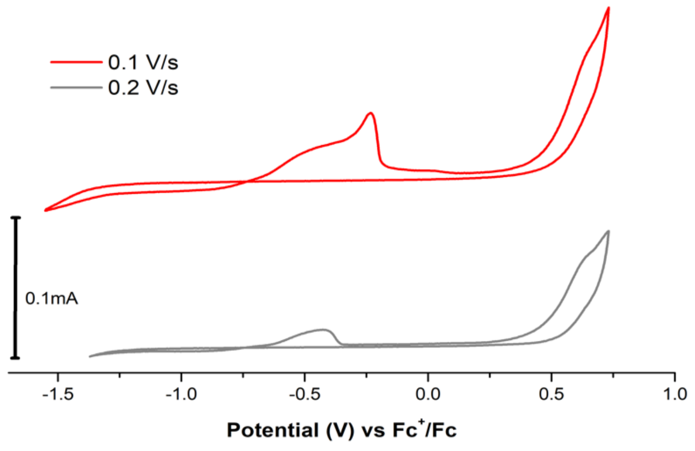

Figure S23. Cyclic voltammetry scans at different scan rates of complex **1** in CH_3CN.
